# Qualitative and quantitative responses to press perturbations in ecological networks

**DOI:** 10.1038/s41598-017-11221-0

**Published:** 2017-09-12

**Authors:** Giulia Giordano, Claudio Altafini

**Affiliations:** 1 Department of Automatic Control and LCCC Linnaeus Center, Lund University, Box 118, SE-221 00 Lund, Sweden; 2 Division of Automatic Control, Department of Electrical Engineering, Linköping University, SE-58183 Linköping, Sweden

## Abstract

Predicting the sign of press perturbation responses in ecological networks is challenging, due to the poor knowledge of the strength of the direct interactions among the species, and to the entangled coexistence of direct and indirect effects. We show in this paper that, for a class of networks that includes mutualistic and monotone networks, the sign of press perturbation responses can be qualitatively determined based only on the sign pattern of the community matrix, without any knowledge of parameter values. For other classes of networks, we show that a semi-qualitative approach yields sufficient conditions for community matrices with a given sign pattern to exhibit mutualistic responses to press perturbations; quantitative conditions can be provided as well for community matrices that are eventually nonnegative. We also present a computational test that can be applied to any class of networks so as to check whether the sign of the responses to press perturbations is constant in spite of parameter variations.

## Introduction

One of the main goals in ecology is to understand the dynamics of communities of interacting species. To this aim, *press perturbation* experiments are carried out: a persistent perturbation is applied to a species in the community, to assess how the density of the various species changes at the new equilibrium^1^. Responses to press perturbations are often difficult to interpret and counterintuitive, due to the fundamental role of indirect effects^2–5^. The community matrix (i.e., the Jacobian matrix of the system of growth equations, evaluated at an equilibrium) only describes direct interactions among species in a community near equilibrium. However, a *j*-species press may affect species *i* through a complex network including direct and indirect interactions (when species *i* and *j* are dynamically coupled through intermediate species). If the perturbation is small enough and the community has a stable equilibrium point, the net effect is given by the negative adjoint of the community matrix^6–13^, whose (*i*, *j*) entry predicts the overall influence of a *j*-species press on species *i*. The negated inverse of the community matrix can be equivalently considered^1–3, 14^, since it has the same sign pattern under stability assumptions. However, the lack of exact knowledge about direct species interactions (namely, the entries of the community matrix), due to empirical limitations, and the huge uncertainties that affect ecological models^15, 16^, often prevent from predicting even the sign of the variation: does the population density at the new equilibrium increase or decrease with respect to the previous equilibrium, or does it remain the same?

Here we show that for some classes of ecological networks, including mutualistic networks^17^, and monotone networks^19–21^, the sign of press perturbation responses can be determined in a purely qualitative manner from the sign pattern of the community matrix, without any information on the numerical value of its entries. For other classes of networks, we propose semi-qualitative or quantitative approaches. A semi-qualitative approach yields sufficient conditions for community matrices with certain sign patterns to admit negated inverses with all nonnegative entries, for some parameter values. We further show that, when a community matrix has only a limited number of negative entries, the responses to press perturbations can possibly be all mutualistic when the community matrix has the property of being eventually nonnegative^22, 23^: this (quantitative) property of Perron-Frobenius type implies that the negative direct interactions only have a transient effect on the dynamics, but leave no trace on the press perturbation response at steady state.

Finally, we present a computational test that exploits the multi-affine structure of the problem to check whether the sign of a press perturbation response is preserved when parameters are uncertain. Such a test can be employed for the analysis of any kind of community (not necessarily mutualistic, but also competitive).

## Results

**Figure 1:**
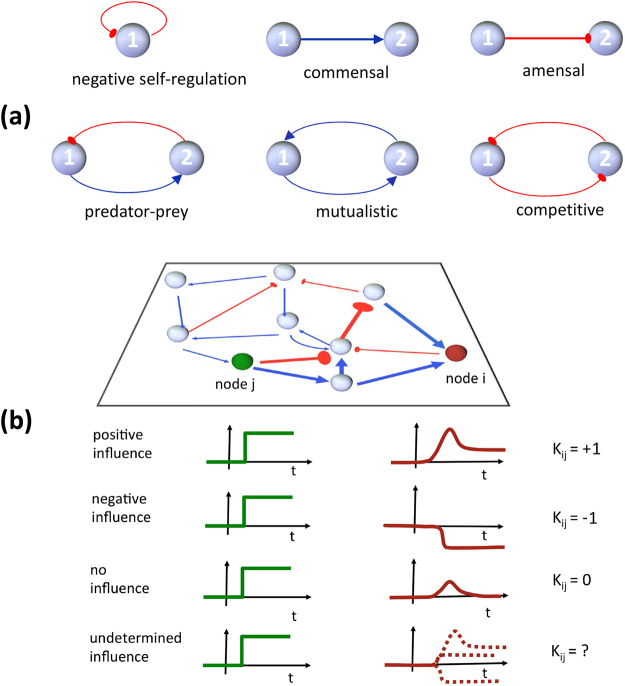
(**a**) Signed edge representations of basic single and pairwise interactions in ecological networks: positive edges are blue, negative edges are red. (**b**) From press perturbation to steady-state influences. Even when *J*_*ij*_ = 0 (no direct effect of species *j* on species *i* in the community matrix), indirect steady-state influences can appear in response to press perturbations applied to node *j*, when *i* and *j* are connected through indirect paths (thick edges). Sometimes the influence has a qualitatively determined sign, and the (*i*, *j*) entry of the influence matrix *K* is sign-definite. When the influence sign is indeterminate, since it depends on the parameter values used in the model, semi-qualitative and quantitative approaches can be used.

Before entering into the details of our contribution, we need to review a few basic concepts about ecological networks. These are compendia of elementary interactions among species in an ecosystem and can be visualised as a graph, whose nodes represent species and whose edges represent interactions. Single and pairwise interactions that normally appear in this context are shown in Fig. 1(a), along with their graph representation. Assembling all of these elementary interactions among *n* species leads to a signed digraph ${\mathscr{G}}(S)$ that represents the whole ecological network, where *S* is a *n* × *n* matrix whose entry [*S*]_*ij*_ is +1 if a positive edge goes from *j* to *i*, −1 if a negative edge goes from *j* to *i*, and 0 otherwise. The dynamics of an *n*-species community can be described by the nonlinear system

1 $$\dot{x}(t)=f(x(t)),$$

where the *i*th component of the vector *x*(*t*) = [*x*_1_(*t*) … *x*_*n*_(*t*)]^Τ^ represents the population density of species *i* and the *i*th component of *f*(*x*(*t*)) = [*f*_1_(*x*(*t*)) … *f*_*n*_(*x*(*t*))]^Τ^ is the corresponding overall growth rate, which is a function of (some or all of) the species densities. We assume that the system admits an asymptotically stable equilibrium point $\bar{x}$, such that $f(\bar{x})=0$. The *community matrix* associated with the system in (1),

2 $$J={\frac{\partial f(x)}{\partial x}|}_{x=\bar{x}},$$

is the Jacobian matrix of the system, evaluated at the equilibrium $\bar{x}$. Its entry *J*_*ij*_ expresses the direct effect of species *j* on the growth rate of species *i*. The sign of the entries of *J* tells us whether a species has a positive/negative direct influence, or no direct influence, on each of the other species, and this is visually represented in the associated graph by a positive/negative edge, or no edge, between the two corresponding nodes. Therefore, there is an equivalence between the overall network graph ${\mathscr{G}}(S)$ and the sign pattern of the community matrix *J*: denoting by sgn(·) the elementwise sign function (sgn(*z*) = +1 if *z* > 0, sgn(*z*) = −1 if *z* < 0, sgn(*z*) = 0 if *z* = 0), we have sgn(*J*) = *S*. We assume that each species has a negative self-loop, representing for instance density-dependent growth rate. This assumption is necessary for the asymptotic stability of the dynamical system in (1) at $\bar{x}$ under the monotonicity assumptions introduced below (however, we will be able to remove this assumption later, when proposing the vertex algorithm that deals with uncertain community matrices). Since we work under asymptotic stability assumptions, it must be *det*(−*J*) > 0 (see e.g. ref. 24), hence *J* is invertible. While *J* includes direct effects only, the net steady-state influence that combines all direct and indirect effects is given by *M* = adj(−*J*), the negative adjoint matrix of the community matrix^6–13^. Its entry *M*_*ij*_ predicts the response of species *i* to a press perturbation on species *j*: if the density of species *j* is experimentally altered and held at a higher level, then, at the new equilibrium, the density of species *i* will be higher if *M*_*ij*_ > 0, lower if *M*_*ij*_ < 0 and unchanged if *M*_*ij*_ = 0, see Fig. 1(b). Since adj(−*J*) = (−*J*)^−1^det(−*J*), and det(−*J*) > 0 in view of stability, we can equivalently consider the sign pattern of −*J* ^−1^, see refs. 1–3,

3 $$K={\rm{sgn}}(-{J}^{-1})={\rm{sgn}}[{\rm{adj}}(-J)],$$

which yields the qualitative effect of all species presses on all other species. We call *K* the *influence matrix*. (Note that *K* can be fully positive, including diagonal entries, even though the diagonal entries of *J* are assumed to be negative). The influence matrix can be determined in principle from field experiments^1^, but this is difficult in practice, especially for large communities^3^. Another approach computes the community matrix *J*, and then the influence matrix, from (1), with parameters based on available data. Yet, parameter values are often uncertain. Can we provide qualitative influence matrices, in spite of the inherent uncertainty in the community matrix?

### Influence matrices: from qualitative to quantitative aspects

Properties that can be investigated simply based on the topology and the signature of the graph ${\mathscr{G}}(S)$ (whenever these features can be comfortably assumed) are referred to as *qualitative*, since they are satisfied by any matrix belonging to the qualitative class *Q*[*S*] of all matrices having the same sign pattern as *S* (zeros included).

In ecological networks, often we know the interaction graph, but we lack quantitative information. Qualitative approaches are then crucial to evaluate the response to press perturbations. Indeed, for a particular class of systems, which includes all mutualistic and monotone networks, the influence matrix can be determined exclusively based on *S* (the sign pattern of the community matrix), without the need of setting values for the *J*_*ij*_. For other classes of systems, semi-qualitative and quantitative approaches can give us useful insights.

#### Monotone systems yield qualitative networks

Monotonicity, intended as a proxy for ordered, oscillation-free dynamical behaviour^19–21^, is a property of paramount importance in a qualitative setting. A system of the form (1), whose Jacobian has negative diagonal entries in view of our assumption that each species has a negative self-loop, is monotone if and only if^19^:

1. Its Jacobian $\frac{\partial f(x)}{\partial x}$ is sign constant everywhere (including at the equilibrium $\bar{x}$), i.e., ∀*x*
  $${\rm{sgn}}(\frac{\partial f(x)}{\partial x})=S;$$
2. There exists a *gauge transformation* Σ (i.e., a matrix with diagonal elements Σ_*ii*_ = ±1 and zero off-diagonal elements^25^) such that Σ*S*Σ is a Metzler matrix (namely, it has nonnegative off-diagonal entries).

**Figure 2:**
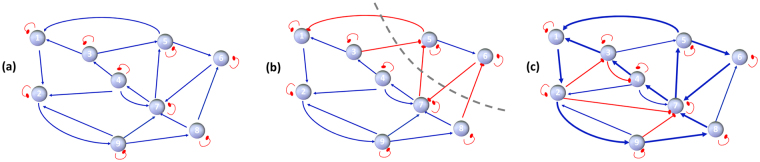
(**a**,**b**) Qualitative networks associated with monotone systems. In (**a**) the system community matrix *J* is Metzler, and so is *S* = sgn(*J*): the graph ${\mathscr{G}}(S)$ has all positive edges (excluding self-loops). Being ${\mathscr{G}}(S)$ strongly connected, *K* > 0 elementwise. In (**b**) the graph ${\mathscr{G}}(S)$ has also off-diagonal negative edges, but all directed cycles are positive: *S* is still associated with a monotone system and *K* is such that Σ*K*Σ > 0, where Σ is a gauge transformation with Σ_55,66_ = −1, Σ_*ii*_ = 1 otherwise (all off-diagonal negative edges are across the cut set shown in gray dashed line). (**c**) Semi-qualitative considerations on signed interaction graphs: given the qualitative class *Q*[*S*], since the graph ${\mathscr{G}}({S}^{+})$ is strongly connected (see, e.g., thick blue lines), matrix −*J* has a positive inverse for some choice of the entries *J*_*ij*_ (with the given sign pattern).

Hence, the Jacobian matrix of a monotone system becomes Metzler after a gauge transformation (see ref. 26 for a similar use of monotonicity and of gauge transformations Σ in the context of social networks). When the matrix *S* is already Metzler (hence Σ = *I*), we have a special type of monotone system, exemplified by the graph in Fig. 2(a). For the more general example of monotone system given by the graph in Fig. 2(b), *S* can be mapped to a Metzler matrix by choosing Σ with Σ_55_ = Σ_66_ = −1 and Σ_*ii*_ = 1 elsewhere. Monotonicity can be immediately detected from the system graph: a system is monotone if and only if all of the cycles in the graph (excluding self-loops) are positive (i.e., have an even number of negative edges). Both graphs in Fig. 2(a) and (b) have this property. Polynomial-time tests on large-scale graphs are described in the literature^25^.

For monotone systems, the influence matrix is always sign definite and can be computed solely based on the qualitative information in *S*, without any knowledge of the edge weights. In particular, the influence matrix associated with a system whose matrix *J* is stable and Metzler has exclusively +1 or 0 entries, and exclusively +1 entries if, in addition, *J* is irreducible (i.e., the graph ${\mathscr{G}}(S)$ is strongly connected, namely, any node can be reached starting from each of the others by following the edges along their direction), as shown in refs. 27, 28 based on ref. 29. Hence, the influence matrix for the example in Fig. 2(a) has *K*_*ij*_ = +1 for all *i*, *j*. For all *mutualistic networks* (ecological networks where all of the interactions are mutually beneficial or commensal), the matrix *S* is Metzler. Hence, stable mutualistic networks always yield nonnegative (and if they are strongly connected, positive) responses to press perturbations. Also for generic monotone systems (whose ${\mathscr{G}}(S)$ can have negative edges, but has positive cycles only), *K* is qualitatively sign determined. In fact, since (Σ*J*Σ)^−1^ = Σ*J* ^−1^Σ, from (3) it is sgn(Σ(−*J*)Σ)^−1^ = Σsgn(−*J* ^−1^)Σ = Σ*K*Σ. Therefore, the same gauge transformation that makes *J* Metzler also affects the signature of the influence matrix *K*. For the example in Fig. 2(b), *K*_*ij*_ = +1 for *i*, *j* ∈ {1, 2, 3, 4, 7, 8, 9} or *i*, *j* ∈ {5, 6} and *K*_*ij*_ = −1 otherwise. As shown by a counterexample in [28, p. 144], *J* (or *S*) being a Metzler matrix is a sufficient, but not a necessary condition for the existence of nonnegative influence matrices: there are systems that have a nonnegative influence matrix even though *J* is not Metzler.

#### Semi-qualitative cases

For a signed graph ${\mathscr{G}}(S)$, monotonicity is lost as soon as a negative cycle of length ≥2 appears (cf. ref. 30). In ecological networks that are not exclusively mutualistic, this will often be the case. Negative cycles of length 2 can originate for instance from predator-prey interactions among two species (see Fig. 1(a)), which are present in abundance in food webs. When monotonicity is lost, purely graphical sign characterisations of the influence matrix *K* are missing. However, *semi-qualitative* conditions can give useful graphical interpretations of what type of interaction graphs have the potential to yield a positive influence matrix: building on results in the literature^31^, we can give a sufficient condition for a system to possibly admit a positive influence matrix (for some values of the community matrix entries) based on the sign pattern of ${\mathscr{G}}(S)$. Precisely, the qualitative class *Q*[*S*] (of all matrices with the same topology and the same signature of *S* = sgn(*J*)) contains at least a community matrix corresponding to a positive influence matrix if the subgraph ${\mathscr{G}}({S}^{+})$, formed by taking only the positive edges of ${\mathscr{G}}(S)$, forms a network-wide strongly connected component. This condition corresponds to the existence of a strongly connected mutualistic “backbone” (a graph ${\mathscr{G}}$ admits a strongly connected mutualistic backbone if the graph obtained from ${\mathscr{G}}$ by removing all negative edges is strongly connected). This means that the (unidirectional) commensal edges and the (bidirectional) mutualistic edges must connect each pair of nodes of the network through directed paths, as in the example in Fig. 2(c).

This semi-qualitative condition can be extended from systems with mutualistic backbone to systems that admit a mutualistic backbone after being transformed by a gauge matrix Σ. For some choice of the values *J*_*ij*_ also these systems have an influence matrix *K* = Σ*O*Σ, where *O* is the matrix of all ones. Networks with predator-prey interactions most often fall into this category (see examples below). The proposed condition guarantees the existence of a community matrix with the desired signed influence matrix within a qualitative class. A method to find such a community matrix based on the quantitative analysis illustrated below will be shown when we discuss the Tatoosh Island example later on. It is worth observing that multiple choices of Σ such that ${\mathscr{G}}{({\rm{\Sigma }}S{\rm{\Sigma }})}^{+}$ is strongly connected are often possible, which implies that there could exist multiple ways to obtain monotone influence matrices *K*. See the Tatoosh Island example below for a more detailed description.

#### Quantitative cases: eventually nonnegative systems

The condition above is semi-qualitative: it does not hold for the whole qualitative class *Q*[*J*], but only for some choices of the weights *J*_*ij*_. To sharpen this characterisation, it is necessary to resort to *quantitative* conditions, which depend on the specific entries of the community matrix *J*. A family of matrices *J* that admit a positive influence matrix (although they are not associated with mutualistic systems) is related to eventually nonnegative matrices^22, 23^. A matrix *B* is eventually nonnegative if it becomes elementwise nonnegative after a certain power: *B* ^*p*^ ≥ 0 for all powers *p* ≥ *p*_*o*_. In practice, even if *B* has some negative entries outside the diagonal, these disappear when taking powers. Hence, in particular, when considering the exponential matrix ${e}^{Bt}={\sum }_{k=0}^{\infty }\frac{{B}^{k}{t}^{k}}{k!}$, the weight of the negative entries of *B* in the infinite sum becomes more and more irrelevant as the time horizon *t* increases. In fact, for any eventually nonnegative matrix *B* such that index_0_(*B*) ≤ 1 (the multiplicity of the eigenvalue 0 of *B* as a root of the minimal polynomial is ≤1), there is always a *t*_*o*_ such that *e* ^*Bt*^ ≥ 0 ∀*t* ≥ *t*_*o*_ [ref. 32, Theorem 3.7]. Eventually nonnegative matrices with a proper diagonal shift lead to community matrices whose negated inverse is elementwise positive. In fact, if we consider an irreducible and eventually nonnegative matrix *B* having spectral radius *ρ*(*B*), then there exists an interval (*ρ*(*B*), *β*) of the real line such that, for all *α* ∈ (*ρ*(*B*), *β*), the matrix *J* = *B* − *αI* is stable and (−*J*)^−1^ > 0, hence *K* > 0 (cf. [ref. 33, Theorem 4.2]). Notice that if *B* is eventually nonnegative, then so is *J* = *B* − *αI*: the diagonal term *αI* plays the same role as the diagonal of a Metzler matrix: it guarantees stability of *J* (which in turn fixes the sign of det(−*J*), leading to (3)). Since *α* > *ρ*(*B*), stability holds regardless of the values on the diagonal of *B*. When seeking in addition *J* that have (−*J*)^−1^ > 0, the presence of an upper bound *β* on the values of *α* implies that the dynamics of *J* cannot be too fast: the dominant eigenvalue of *J* (which is also called the Perron-Frobenius eigenvalue and is equal to *ρ*(*B*) − *α*) is real, negative and small, thus the corresponding mode has a long time constant. Being *J* stable, this means that, in order to have a positive influence matrix, alignment along the dominant direction (determined by the eigenvector relative to the Perron-Frobenius eigenvalue) after a press perturbation must occur slowly enough. When the dominant mode is sufficiently slow, the influence of the negative edges on the dynamics tends to fade away with respect to the positive “backbone” ${\mathscr{G}}({J}^{+})$, hence it does not appear in *K*. When instead *α* > *β*, then the alignment along the dominant direction becomes too fast, and the indirect influence exerted by the negative edges of ${\mathscr{G}}(J)$ cannot be absorbed by the positive backbone ${\mathscr{G}}({J}^{+})$. This condition can be extended to community matrices that are eventually nonegative after a gauge transformation.

### A general algorithm to deal with uncertain community matrices

For a wide class of systems (admitting the so-called *BDC*-decomposition^27, 34, 35^, which includes all systems with a sign-definite Jacobian $\frac{\partial f(x)}{\partial x}$, see SI for details), the qualitative effect of press perturbations on steady-state species densities can be assessed regardless of the chosen parameter values^27^. With an analogous approach, we can check whether the influence matrix remains the same *in spite of parameter variations in bounded intervals*. We consider a generic system (1) under asymptotic stability assumptions: in this case, the system can be asymptotically stable at $\bar{x}$ even though some diagonal entries of *J* are not negative, and the proposed algorithm can still be applied. When the entries of the community matrix *J* are known to belong to an interval, ${J}_{ij}\in [{J}_{ij}^{-},{J}_{ij}^{+}]$, we can assess the sign of the entries of the influence matrix for all possible values of the parameters within the resulting hyper-rectangle in the parameter space by computing it only at the vertices of the hyper-rectangle to which the *J*_*ij*_’s are known to belong. A sign determined influence is identified whenever, upon a press perturbation, the ensuing variation of the considered steady-state value has the same sign as the press (positive influence), the opposite sign (negative influence), or is zero (perfect adaptation^36–38^), *for any possible choice* of the *J*_*ij*_’s within the given intervals, see Fig. 1(b); conversely, if the outcome depends on the choice of the *J*_*ij*_’s (within the bounds), the influence is indeterminate. The numerical vertex procedure provides the determined sign of the influence, or warns us that it is indeterminate (more details on the algorithm are in the Methods section; see also the SI file, where the method is further discussed and applied to some of the examples of ecological networks discussed below). Importantly, the proposed vertex algorithm not only tells us whether an entry of the influence matrix has a constant sign in the whole parameter space, but also provides the maximum and the minimum value that the entry can achieve, given the known uncertainty bounds (even when the influence turns out to have an undetermined sign). Hence, it can be employed to quantify phenomena, also in the presence of uncertain entries.

### Examples

#### Mutualistic networks

By construction, mutualistic networks are ecological networks for which the corresponding community graph contains positive edges only (excluding self-loops). Mutualistic networks are widespread in nature (they portrait, for instance, plant-animal and plant-pollinator interactions) and have been shown to exhibit diverse and complex topologies (nested, hierarchical, compartimentalised, bipartite, etc. refs. 17, 18). Regardless of the topology and of the complexity, the community matrix of these networks is Metzler. Self-limitation on all species is necessary for stability. Then, if stability can be assumed, the influence matrix is automatically nonnegative, or positive if the network is strongly connected. In the latter case, indirect mutualism is always guaranteed, even among non-directly connected species.

#### Plankton-bacteria-protozoa community

**Figure 3:**
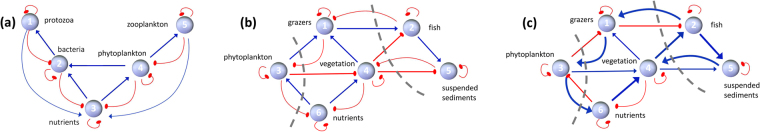
(**a**) Plankton-bacteria-protozoa community^9, 39^. The graph ${\mathscr{G}}({S}^{+})$ is strongly connected, hence the community potentially exhibits indirect mutualism. (**b**,**c**) Indirect competition: the shallow lake community^40^ is strongly connected through a positive cycle that, however, involves negative edges. A suitable gauge transformation Σ, applied through the cut sets shown in panel (b), yields the sign-transformed graph of panel (c), with sign matrix *S*′ = Σ*S*Σ, where now ${\mathscr{G}}({(S^{\prime} )}^{+})$ is strongly connected.

The plankton community from refs. 9, 39 shown in Fig. 3(a) is a classical food-web example of how apparently paradoxical effects can be interpreted in terms of indirect interactions. Phytoplankton under nutrient stress stimulates (through the release of extracellular organic carbon) the production of bacteria. Since bacteria and phytoplankton compete for the same inorganic nutrients, this behaviour seems counterintuitive if we just look at the direct interactions shown in Fig. 3(a). Indirect effects can however clarify this behaviour, see refs. 9, 39 for an analysis. With the tools developed in this paper we can show that, since ${\mathscr{G}}(S)$ contains a strongly connected subgraph composed of positive edges only, the network can even have a fully mutualistic behaviour. In fact, as detailed in the SI, for suitable values of the community matrix entries *J*_*ij*_, the influence matrix *K* is elementwise positive. Such a community matrix has the form *J* = *B* − *αI* with *B* eventually positive, meaning that the positive effects dominate and annihilate the negative ones for times long enough. In this example, it is also possible to build a convex region of parameter uncertainties around such nominal *J*, region in which the network is guaranteed to have nonnegative responses to press perturbations, see SI.

#### Shallow lake community

The example from ref. 40 (see also ref. 41) is reproduced in Fig. 3(b). The system is not monotone (it has several predator-prey cycles), and ${\mathscr{G}}({S}^{+})$ is not strongly connected. However, a gauge matrix Σ such that (Σ*S*Σ)^+^ is irreducible can be found, associated with a partition of the nodes into two groups, see Fig. 3(c). *Phytoplankton*, *fish* and *suspended sediments* (linked by positive or doubly negative paths) are on one side of the partition, while *grazers*, *vegetation* and *nutrients* are on the other side. For some parameter values (as shown in the SI), each of the species in one group induces indirect competition on each of the species in the other group, in response to press experiments.

#### Random Erdös-Rényi networks and eventual nonnegativity

**Figure 4:**
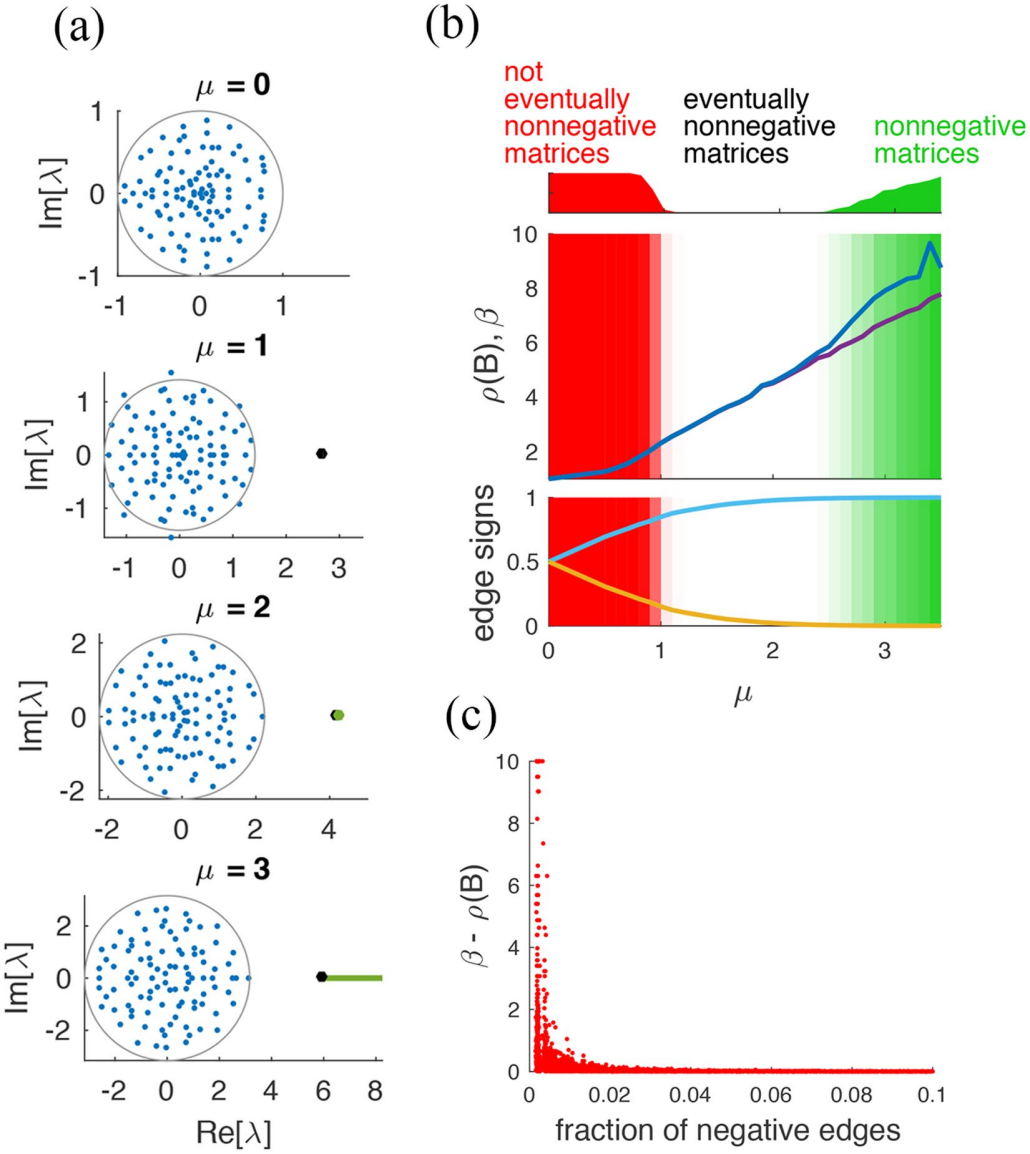
(**a**) From top to bottom: location of the eigenvalues of *B* as *μ* grows, for an ER network of size *n* = 100. When *μ* > 0, *ρ*(*B*) (black dot) detaches from the disk of eigenvalues and moves to the right. The interval (*ρ*(*B*), *β*), shown in green, becomes visible when *μ* > 2. (**b**) Middle panel: The mean value (over 200 realisations) of *ρ*(*B*) (violet) and *β* (blue) as *μ* varies. For *μ* > 2 the interval (*ρ*(*B*), *β*) becomes visible. The red shaded area corresponds to the fraction of realisations of *B* which are not eventually nonnegative (full colour is 100% of realisations, top panel). The green shaded area is the fraction of realisations of *B* that are nonnegative (nearly-full colour on the rightmost part corresponds to 90% of realisations). Bottom: The average fraction of positive (cyan) and negative (yellow) edges in *B* as *μ* varies. Eventually nonnegative matrices start to appear when the negative edges of *B* are less than 15%. (**c**) The width of the interval (*ρ*(*B*), *β*) is shown versus the fraction of negative edges in the 200 realisations. The points are upper bounded by a hyperbolic curve.

Our next example is based on randomly generated graphs. We are interested in finding eventually nonnegative matrices *B* and values *α* ∈ (*ρ*(*B*), *β*) such that the community matrix *J* = *B* − *αI* is (eventually nonnegative, and) stable and such that (−*J*)^−1^ > 0. Once *B* is given, computing *α* is a simple application of the vertex algorithm (Theorem 4) with uncertainty only on the diagonal entries of *J*: *J*_*ii*_ ∈ (*ρ*(*B*) − *β*, 0). Here the matrices *B* are obtained as adjacency matrices of directed random networks of Erdös-Rényi (ER) topology, with edge probability *p* = 0.05 and edge weights drawn from a normal probability distribution ${\mathscr{N}}(\mu ,\mathrm{1/(}pn))$, *n* = 100 (see refs. 42, 43). If *μ* = 0, Girko circle law ensures that, when *n* → ∞, the eigenvalues are inside the unit disk with probability 1; hence, the adjacency matrices *B* are basically never eventually nonnegative. However, when the mean *μ* passes from 0 to a positive value, we introduce a positive bias in the edge weights, and the ratio between the number of positive and negative coefficients increases, see Fig. 4(b). The effect of *μ* > 0 on the eigenvalues location is that *n* − 1 of the eigenvalues are contained in a disk of radius $\sqrt{1+{\mu }^{2}}$, while the *n*-th eigenvalue is to the right of the disk^42^. This is the Perron-Frobenius eigenvalue *ρ*(*B*): it is real, positive, simple and equal to the spectral radius of *B*, see Fig. 4(a). The value of *ρ*(*B*) grows linearly with *μ*, see Fig. 4(b). When *μ* > 1, some matrices *B* become eventually nonnegative, although the interval (*ρ*(*B*), *β*) is very small. Only when *μ* > 2 the interval becomes appreciable, and the set of matrices *J* = *B* − *αI*, *α* ∈ (*ρ*(*B*), *β*) significant. When *μ* > 2.5, some of the matrices *B* become nonnegative (and hence *J* Metzler). When *μ* = 3.5, around 90% of all *B* are nonnegative. If the width *β* − *ρ*(*B*) of the interval in which *J* is stable and (−*J*)^−1^ > 0 is plotted against the fraction of negative edges in *B*, then a curve evocative of an inverse relationship emerges, see Fig. 4(c). The hyperbola delimits the convergence rates that a stable community matrix is allowed to have if it has to preserve the positivity of the influence matrix. The region delimited by the hyperbola, in fact, describes how bigger the real part of the dominant eigenvalue of *J* (i.e., the Perron-Frobenius eigenvalue, sometimes called asymptotic resilience^44^) can be with respect to the spectral radius *ρ*(*B*). When *β* = *ρ*(*B*), hence *α* = *ρ*(*B*), matrix *J* is only marginally stable, i.e., it lies on the boundary of its stability region. When *β* > *ρ*(*B*), for *α* ∈ (*ρ*(*B*), *β*), *J* = *B* − *αI* has a dominant eigenvalue (the one with highest spectral abscissa) inside the left half of the complex plane: the higher is *α*, the faster is the convergence of *J*.

#### A large-scale signed network example: Tatoosh Island

**Figure 5:**
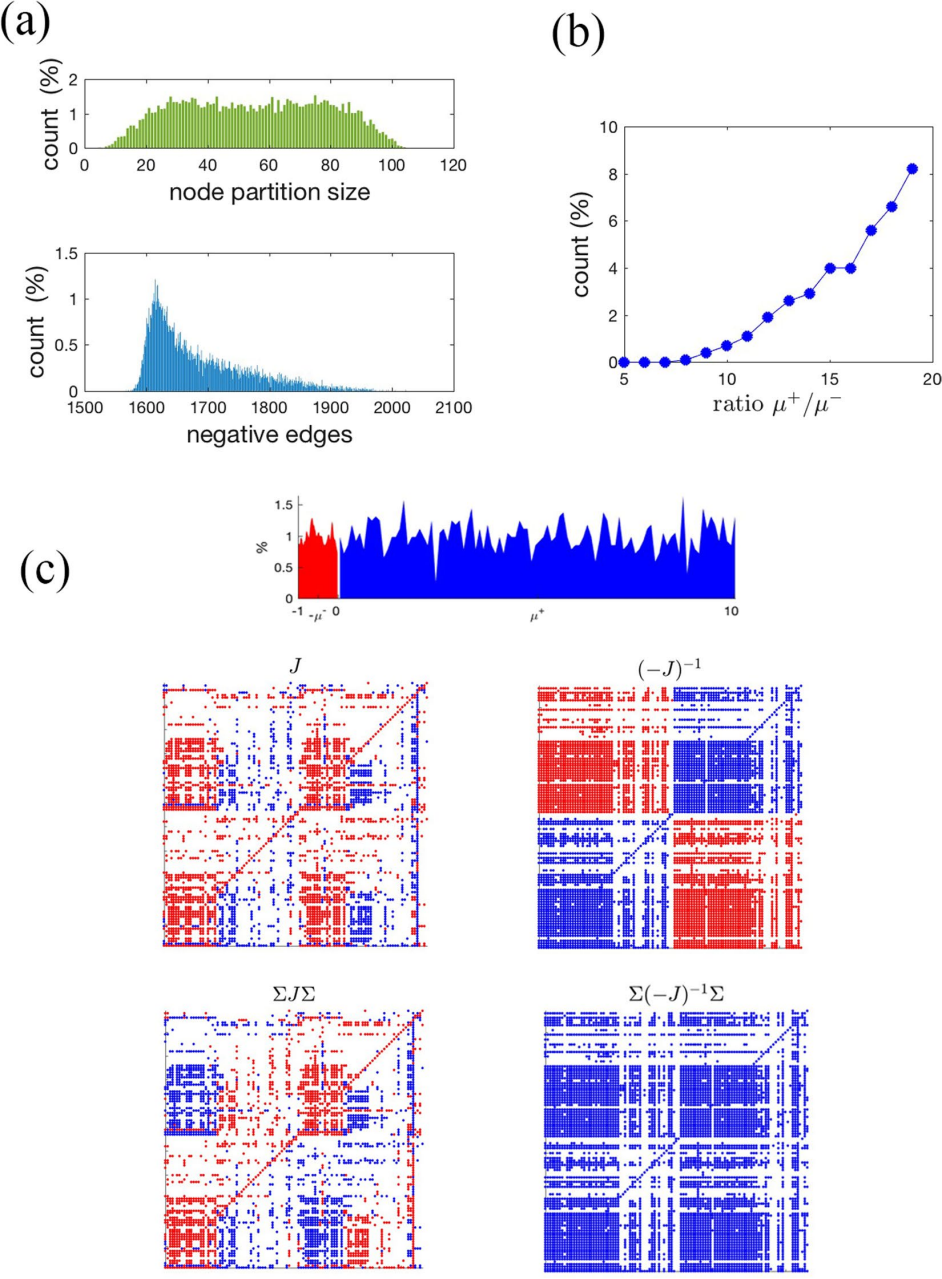
Tatoosh Island example. (**a**) Upper histogram: “node partition size” refers to the fraction of − signs in 10^4^ gauge transformations Σ yielding a strongly connected ${\mathscr{G}}(({\rm{\Sigma }}S{\rm{\Sigma }}{)}^{+})$. The range 5 ÷ 104 is explored. Lower histogram: corresponding number of negative edges in Σ*S*Σ for the 10^4^ choices. (**b**) When the positive edges are drawn from a uniform probability distribution of mean *μ* ^+^ and the negative edges from one of mean *μ* ^−^, then for *μ* ^+^/*μ* ^−^ ≥ 8 community matrices *J* such that Σ*J*Σ are eventually nonnegative start to appear. The percentage of samples *J* with such a property grows with *μ* ^+^/*μ* ^−^. (**c**) A specific choice of Σ and edge weights with *μ* ^+^/*μ* ^−^ = 10. Histogram: edge weights distributions for *J* (red for − and blue for +). The 4 scatter plots represent the edge signs (again red for − and blue for +) of *J* (upper left), Σ*J*Σ (lower left), (−*J*)^−1^ (upper right) and Σ(−*J*)^−1^Σ (lower right). The latter has all positive entries (dots are missing when the edge weight is <10^−4^ in absolute value). Hence (−*J*)^−1^ is monotone.

To describe the intertidal interaction network of Tatoosh island, including a food web and other non-trophic interactions that are mutualistic, competitive, commensal and amensal, ref. 45 proposes a network that involves 110 species in 3096 interactions (1087 ‘+’ edges, 2009 ‘−’ edges). The network is downloadable from the Dryad Digital Repository http://dx.doi.org/10.5061/dryad.39jv1. Negative self-regulation coefficients are added on the diagonal of the signed matrix *S* (the original dataset already has 70 negative self-regulation coefficients). The resulting graph ${\mathscr{G}}(S)$ is strongly connected. Its positive subgraph ${\mathscr{G}}({S}^{+})$ has a very large strongly connected component involving 100 of the 110 species. The excluded taxa are at the top or at the bottom of the food chain for the considered network: top predators (such as *Haliaeetus leucocephalus*, *Falco peregrinus* and *Henricia*) have no outgoing positive edges, while prey at the bottom (*Corallina vancouvriensis*, *Diatoms*, *Articulated corralines* and *Phytoplankton*) have negative incoming edges only, hence they cannot be involved in directed cycles in ${\mathscr{G}}({S}^{+})$. Consequently, our condition for the existence of a mutualistic influence matrix (Theorem 2 below) is not satisfied. However, if we focus on monotone, rather than mutualistic, influence matrices, it is easy to see that with the exclusion of a single species (*Falco peregrinus*, which is connected to the rest of the network via a single predator-prey interaction with *Corvus caurinus*, and hence can never be part of a strongly connected subgraph of only positive cycles), on the network of remaining 109 species there are many possible ways to choose a gauge transformation Σ so that ${\mathscr{G}}(({\rm{\Sigma }}S{\rm{\Sigma }}{)}^{+})$ is strongly connected. So, if we focus on the subnetwork of size *n* = 109, Theorem 2 guarantees that for each such Σ there exists a monotone influence matrix, i.e., *K* such that Σ*K*Σ is a matrix of all 1. The number of possible choices of Σ is 2^109^, far out of reach of any exhaustive search. In Fig. 5(a) we explore 10^4^ gauge transformations which result in ${\mathscr{G}}(({\rm{\Sigma }}S{\rm{\Sigma }}{)}^{+})$ strongly connected. As can be seen on the top histogram, they span a broad range of possible +/− partitions in Σ, and correspond to a number of negative edges in Σ*S*Σ which is between 1600 and 2000 (lower histogram). Clearly with such a large fraction of negative edges, if we select all edge weights from the same probability distribution we cannot hope to obtain a *J* which is eventually nonnegative. However, if we decrease the importance of the negative edges, for instance drawing them from a probability distribution of lower mean, then it is easy to obtain sampled (and stable) *J* such that Σ*J*Σ is eventually nonnegative and hence Σ(−*J*)^−1^Σ positive. In Fig. 5(b,c), the positive edges are drawn from a uniform distribution of mean *μ* ^+^ and the negative ones from a uniform distribution of mean *μ* ^−^. When the ratio *μ* ^+^/*μ* ^−^ ≥ 8, then samples having Σ(−*J*)^−1^Σ > 0 start to appear. When *μ* ^+^/*μ* ^−^ ≥ 15, then around 4% of the samples satisfy this property (Fig. 5(b)). An example with *μ* ^+^/*μ* ^−^ = 10 is shown in Fig. 5(c). By looking at *S* alone, it is impossible to ascertain that such a network admits a choice of edge weights *J* whose negated inverse is monotone. By combining our semi-qualitative analysis with the computational test given by eventual nonnegativity, the verification is however very easy.

## Discussion

A vast fraction of the literature on ecological networks deals with models that consider only a specific type of interaction: mutualistic, trophic, parasitic, etc. Yet, real ecological networks involve interactions of different types, leading to community graphs in which mutualism, antagonism, commensalism and competition, etc. coexist^45–48^. Hence, the community dynamics can be significantly more complex^47, 48^, and the field experiments more difficult to interpret. Given that applying environmental perturbations is one of the most common ways to gather experimental data on ecological systems, being able to classify in a qualitative sense experiments such as press perturbations becomes a key factor, for instance, for empirical community graph inference. Qualitative methods and signed digraphs have been used in ecology since the Seventies^6–8^, although mostly for investigating stability of an ecosystem. In the context of this paper, stability is always assumed, which considerably simplifies the qualitative analysis of press perturbation responses. In fact, when stability is missing, the sign of det(−*J*) is not a priori known, and the sensitivity of some variables to presses may grow unbounded.

When features like indirect interactions are investigated, the qualitative methods used in the literature are mostly inspired by loop analysis^8, 11, 13, 49^, i.e., decompositions of the determinant of *J* into products of ‘elementary circuits’. The products of the signs of these circuits gives the sign of the sensitivity, i.e., of the steady-state influence matrix *K*. The drawback of this approach is that computationally it explodes at very modest sizes^13^, hence it is inapplicable for large-scale ecological networks; another drawback of loop analysis is the sign ambiguity of the resulting press perturbation responses. One way to cope with such indeterminacy, discussed in ref. 15, is to sample the parameter space and compute statistics of the ensuing signs. Other approaches exist in the literature to qualitatively estimate press responses. For instance in ref. 50 the influence coefficients *K*_*ij*_ are computed by aggregating together all (suitably weighted) paths between nodes *j* and *i*. More organic overviews of the topic are available in refs. 13, 49, 51.

The methods proposed in this paper for this task are meant to provide a classification of the possible cases:

1. Those in which a purely qualitative analysis is possible (mutualistic and monotone networks);
2. Those in which a proof of existence is possible (*S* such that ${\mathscr{G}}({S}^{+})$ or ${\mathscr{G}}({({\rm{\Sigma }}S{\rm{\Sigma }})}^{+})$ is strongly connected).
  In addition, they are meant to propose
3. A class of matrices (not of qualitative nature, but endowed with the “strong” properties of Perron-Frobenius theorem) which also guarantee positivity of the influence matrix;
4. An algorithm able to treat any kind of interval matrices, useful whenever uncertainty bounds on the entries of *J* are available or are sought.

Given the long history of the problem of inverse positivity in linear algebra^31, 52, 53^ we expect this classification to be a nearly-complete corpus of conditions under which the problem of determining the signs of the influence matrix is solvable.

Several byproducts of our analysis should also be of interest to the Ecological networks community. For instance it follows from the properties of inverse positive matrices that if a mutualistic network is strongly connected, then its influence matrix is positive and *full*: all species of the ecosystem have a positive sensitivity to press perturbations on any of the species. Our analysis shows also that in turn, when we aim at inferring the community graph from press perturbations, strong connectivity of a mutualistic community implies that press experiments alone cannot provide important information such as edge density (intended as number of edges that *J* should have): irreducible, Metzler matrices *J* that lead to *K* > 0 may have as few as *n* + 1 or as many as *n* ^2^ − *n* (off-diagonal) edges.

When a community network has a mixture of positive and negative edges, then the corresponding *S* ^+^ may fail to be strongly connected, which reflects a smaller “likelihood” of steady-state mutualism to occur. The prototype of this topology is the predator-prey interaction in Fig. 1(a), a motif at the core of food webs: overabundance of predatory-prey cycles in an ecological network renders less plausible the possibility that press responses yield positive indirect effects over the whole network. However, this does not rule out an analysis based on the tools presented in this paper. Only, rather than mutualistic press responses one has to expect signed responses. In monotone networks the graph is always partitionable into two mutualistic subcommunities connected by negative edges only, as in Fig. 3(b). Given that such a partition is found also in the influence matrix, it is in principle observable through field experiments such as press perturbations.

Just like mutualism can be generalised to monotonicity, also the proposed semi-qualitative and quantitative conditions can be extended to ensure that *K* is positive after a gauge transformation. Gauge transformations do not alter the sign of the cycles of *K* (or *J*), hence do not change the fraction of predator-prey loops. Applying gauge transformations, strongly connected (Σ*S*Σ)^+^ normally exist, and hence the signs of the indirect interactions can still be uncovered from those of the community matrix, as we did in the shallow-lake community example, cf. Fig. 3(b,c) and in the Tatoosh Island example, see Fig. 5.

It is worth emphasizing another use of our semi-qualitative sufficient conditions in this context: screening for strongly connected subgraphs ${\mathscr{G}}({({\rm{\Sigma }}S{\rm{\Sigma }})}^{+})$ corresponds to screening for orthants (i.e., signatures Σ) in which the influence matrix can have “support”. In principle this information can be of interest also when one is seeking to express the probability of finding the influence matrix in the various orthants, for instance through a sampling of the parameter space^15^. As the number of possible sign patterns of the influence matrix explodes with the network size, knowing a priori where this probability can be localised seems to us a useful information.

Unlike monotone matrices, eventually nonnegative matrices do not form a qualitative class, but rather a set in parameter space with boundaries difficult to characterise explicitly. In addition, also the time constant of the eigenvalues of *J* plays an important role in inverse positivity of eventually nonnegative matrices: increasing the convergence speed (i.e., moving the eigenvalues deeper in the left half of the complex plane) may lead to a loss of mutualism in the influence matrix. The hyperbolic shape of the region in Fig. 4(c) suggests that, even for *J* eventually nonnegative, a moderately high convergence speed can lead to indirect steady-state influences that are all positive in the network only when there are few off-diagonal negative direct interactions in *J* (or many, but all small). When the number of negative direct interactions grows, even if *J* is eventually nonnegative, the response of the system to perturbations has to be slow enough to attain indirect mutualism. A slow dynamics, however, requires having a community matrix *J* that lies near the edge of its stability region. In other words, for communities that are near-mutualistic, different steady-state fates are possible, depending on the dominant time constant: when the dynamics is fast, it is unlikely that a community yields a fully mutualistic steady-state influence matrix; when instead the community dynamics evolves slowly enough, near its stability boundary, this may lead to purely mutualistic steady-state indirect interactions. Analogous considerations can be formulated if instead of the single dominant mode, some average over the real part of all the eigenvalues of *J* is considered^44^. It is tempting to interpret this speed-dependent phenomenon as a tipping point condition, which gets triggered when a naturally slow process undergoes a rapid acceleration.

A limitation of quantitatively computing the inverse of the negated community matrix is that it is highly sensitive to numerical values of the entries *J*_*ij*_, see refs. 2, 13. By isolating classes of matrices, such as eventually nonnegative matrices, and even more by constructing numerical tests valid for polytopes of community matrices (such as the proposed vertex test), we provide ways to bypass the indeterminacy in the computation of the inverse community matrix. In particular, the proposed vertex algorithm allows us to check if the response to a press perturbation has the same sign *for all possible community matrices having their entries in given intervals*, hence within a hyper-rectangle in the parameter space, by computing its sign on the vertices of the hyper-rectangle only. For a network of size *n*, the number of community matrices to be tested is ${2}^{{n}^{2}}$ in the worst case, which is however very unlikely to occur in ecological networks, where each species typically interacts with just a few other species, regardless of the size of the network. This sparsity is beneficial, because the algorithm complexity scales exponentially with the number of uncertain *nonzero entries* in the community matrix. In particular, let *c* denote the fraction of nonzero interactions among the *n* species, so that we have approximately *cn* ^2^ nonzero entries in the community matrix. We can reasonably assume that, when *n* grows, *c* decreases as 1/*n* (see, e.g. refs. 54–56), since each species interacts with a few of the others only, and the number of interactions per species saturates to a constant (and relatively small) value *p*. The number of nonzero entries then grows linearly (and not quadratically) with *n* and and we can expect to test 2^*pn*^ matrices. Sparsity could be additionally exploited by adopting strategies along the lines of the tree-like algorithm discussed in ref. 27. In any case, the computational effort is paid back by a very strong knowledge: if a qualitative answer is provided for an entry, then the influence will have the same sign *for all possible points in the considered parameter space*. If the sign is not the same for all possible points, still it might be the same for almost all possible points, or with high probability: in this case, our results are nicely complemented by the probabilistic approach in ref. 14, whose exact formulas quantify the probability of obtaining a qualitatively wrong prediction in the presence of uncertainties that follow a probability distribution, providing valuable additional insight.

## Methods

The technical methods overviewed below are presented in a more complete form in the SI file, with detailed proofs of the results as well as additional information.

### Qualitative methods

Given a matrix $A\in {{\mathbb{R}}}^{n\times n}$, ${\mathscr{G}}(A)$ denotes the digraph with adjacency matrix *A*, while *Q*[*A*] is the *qualitative class* of all matrices having the same sign pattern as *A*. In particular, *Q*[*A*] always contains a signature matrix *S* = sgn(*A*) whose entries are in {0, −1, +1}. Clearly, ${\mathscr{G}}(A)$, ${\mathscr{G}}(S)$ and ${\mathscr{G}}(B)\,\forall B\in Q[A]$ all have the same (signed) graph, but possibly different numerical weights.

#### Monotone dynamical systems

Denote by *x*(*t*) the solution of (1) at time *t* with initial condition *x*(0). Consider a diagonal matrix Σ = diag(*σ*) with diagonal entries *σ* = (*σ*_1_, …, *σ*_*n*_), *σ*_*i*_ ∈ {±1} (called gauge matrix). The vector *σ* identifies a partial order for the *n* axes of ${{\mathbb{R}}}^{n}$, which can be the “natural” one when all *σ*_*i*_ = +1, or the opposite when all *σ*_*i*_ = −1. The system in (1) is said *monotone* with respect to the partial order *σ* if, for all initial conditions *x*_1_(0), *x*_2_(0) such that Σ*x*_1_(0) ≤ Σ*x*_2_(0), it is Σ*x*_1_(*t*) ≤ Σ*x*_2_(*t*) ∀*t* ≥ 0, see refs. 19–21. The system in (1) is said *strongly monotone* with respect to the partial order *σ* if, for all initial conditions *x*_1_(0), *x*_2_(0) such that Σ*x*_1_(0) ≤ Σ*x*_2_(0), *x*_1_(0) ≠ *x*_2_(0), it is Σ*x*_1_(*t*) < Σ*x*_2_(*t*) ∀*t* > 0. Monotonicity of a system can be checked in terms of its Jacobian $J(x)=\frac{\partial f(x)}{\partial x}$ based on the Kamke condition [19, Lemma 2.1]: the system in (1) is monotone w.r.t. the order *σ* if and only if

4 $${\sigma }_{i}{\sigma }_{j}{J}_{ij}(x)\ge 0\quad \forall x\in {{\mathbb{R}}}^{n},\quad \forall i,j=1,\ldots ,n\quad i\ne j,$$

or, in matrix form, Σ*J*(*x*)Σ is Metzler $\forall x\in {{\mathbb{R}}}^{n}$. This condition implies that *J*(*x*) must have the same signature *S* = sgn(*J*(*x*)) everywhere, hence it can be stated equivalently in terms of *S* as

5 $${\sigma }_{i}{\sigma }_{j}{S}_{ij}\ge 0\quad \forall i,j=1,\ldots ,n\quad i\ne j\mathrm{.}$$

The condition in (5) admits a graph-theoretical reformulation. The system in (1) is monotone with respect to some order if and only if all directed cycles of length >1 of the signed digraph ${\mathscr{G}}(S)$ (or ${\mathscr{G}}(J)$) have positive sign. A matrix *M* is said *irreducible* if no permutation matrix *P* exists such that

$${P}^{\top }MP=[\begin{array}{cc}{M}_{1} & 0\\ {M}_{2} & {M}_{3}\end{array}]$$

with *M*_1_ and *M*_3_ square matrices; equivalently, ${\mathscr{G}}(M)$ is strongly connected. Clearly, if *M* is irreducible, any matrix *A* ∈ *Q*[*M*] is irreducible, since ${\mathscr{G}}(M)$ and ${\mathscr{G}}(A)$ have the same topology and the same edge signs. Monotonicity, combined with irreducibility of *J*(*x*) at all *x*, implies strong monotonicity of (1).

### Semi-qualitative methods

Let *A* ^+^ be the nonnegative part of *A*,

$${A}_{ij}^{+}=(\begin{array}{cc}{A}_{ij} & {\rm{if}}\,{A}_{ij}\ge 0\\ 0 & {\rm{if}}\,{A}_{ij} < 0\end{array}$$

and $\hat{A}$ the following “lifting” of *A* to ${{\mathbb{R}}}^{2n\times 2n}$ (see e.g. ref. 52):

$$\hat{A}={[\begin{array}{cc}0 & A\\ -{A}^{\top } & 0\end{array}]}^{+}\mathrm{.}$$

A matrix *A* is *fully indecomposable* if no permutation matrices *P*_1_, *P*_2_ exist such that

$${P}_{1}A{P}_{2}=[\begin{array}{cc}{A}_{1} & 0\\ {A}_{2} & {A}_{3}\end{array}]$$

where *A*_1_ and *A*_3_ are square matrices. The matrix *A* is fully indecomposable if and only if, for some permutation matrix *P*, *PA* is irreducible and has nonzero diagonal entries (see for instance [53, p.56]). We have the following theorem by Fiedler and Grone^31^.

**Theorem 1** *Given a fully indecomposable signature matrix S*, *there exists a matrix B* ∈ *Q*[*S*] *such that B* ^−1^ > 0 *if and only if the matrix* $$\hat{S}={[\begin{array}{cc}0 & S\\ -{S}^{\top } & 0\end{array}]}^{+}$$ *is irreducible*.

This equivalence condition can be useful as a tool for preliminary screening, to immediately understand if a matrix with a given signature cannot have a positive inverse. Based on results in ref. 31, we can get qualitative sufficient conditions that allow a matrix with a given sign structure to have a negative inverse with positive entries.

**Theorem 2** *Given an irreducible S*, *with S*_*ii*_ = −1 ∀*i* = 1, …, *n*, *if S* ^+^ *is irreducible*, *then there exists J* ∈ *Q*[*S*] *such that* −*J* ^−1^ > 0.

By means of counterexamples, it can be shown that the condition is sufficient, but not necessary: there are irreducible sign patterns *S*, with *S* ^+^ reducible, for which ∃ *J* ∈ *Q*[*S*] such that −*J* ^−1^ > 0. Still, Theorem 2 provides useful intuition on the existence of a positive backbone in networks that can admit a fully positive influence matrix, giving us insight into the design principles rooted in the interaction pattern.

### Quantitative methods: eventually nonnegative systems

A matrix $M\in {{\mathbb{R}}}^{n\times n}$ is *eventually nonnegative* if $\exists \,{p}_{0}\in {\mathbb{N}}$ such that, ∀*p* ≥ *p*_0_, *M* ^*p*^ ≥ 0 elementwise; equivalently, its spectral radius

$$\rho (M)=\mathop{{\rm{\max }}}\limits_{{\lambda }_{i}\in \sigma (M)}|{\lambda }_{i}|$$

is a real, positive eigenvalue of *M*, called the Perron-Frobenius eigenvalue, and the corresponding left and right eigenvectors are elementwise nonnegative. Denote by index_*λ*_(*M*) the multiplicity of the eigenvalue *λ* of *M* as a root of the minimal polynomial (*i*.*e*., the dimension of the largest Jordan block associated with *λ*). Then we have the following result, adapted from [ref. 33, Theorem 4.2].

**Theorem 3** *Consider J* = *B* − *αI*, *where* $B\in {{\mathbb{R}}}^{n\times n}$ *is irreducible and eventually nonnegative*, *with* index_0_(*B*) ≤ 1. *Then*, ∃*β* > *ρ*(*B*) *such that* ∀*α* ∈ (*ρ*(*B*), *β*), −*J* = *αI* − *B has a positive inverse*.

More generally, if ∃ *α* such that *J* + *αI* = *B* is eventually nonnegative and satisfies Theorem 3, then the influence matrix derived from *J* is positive: (−*J*)^−1^ > 0, hence *K* > 0 elementwise. Notice that *B* eventually nonnegative implies *J* eventually nonnegative (but with different time constants). Note that the converse of Theorem 3 is not true. Other closely related cases are described in ref. 57.

### Compute the influence matrix from an uncertain community matrix

Given $x\in {{\mathbb{R}}}^{n}$, we consider the nonlinear system

6 $$\dot{x}(t)=f(x(t))+Eu(t),\quad y(t)=Hx(t),$$

where *f*(·) is continuously differentiable, $u\in {\mathbb{R}}$ is an input, $y\in {\mathbb{R}}$ is an output, and we assume that there exists an asymptotically stable equilibrium point $\bar{x}$. Then, both the state asymptotic value $\bar{x}(u)$ and the output asymptotic value $\bar{y}(u)=H\bar{x}$ are functions of *u*. The *steady-state input-output influence* ^27^ is the ensuing variation of the steady state of the system output *y*, upon a variation in the input *u* (a relevant variable or parameter). We assume that the considered input perturbation is small enough to ensure that the stability of $\bar{x}(u)$ is preserved (being the eigenvalues of the Jacobian matrix continuously dependent on the entries, which are in turn continuous functions of *u*). Of course, different variables of interest for the system may respond with a steady-state variation that has the same sign as the input variation, the opposite sign, or is zero. The steady-state input-output influence is *qualitatively signed* if it always has the same sign (positive, negative, or zero), regardless of the choice of parameter values^27^. Denoting by *J* the community matrix, in view of the implicit function theorem, the input-output influence (or sensitivity) can be expressed as^27^

7 $$\frac{\partial \bar{y}}{\partial \bar{u}}=H{(-J)}^{-1}E=\frac{{\rm{\det }}[\begin{array}{cc}-J & -E\\ H & 0\end{array}]}{{\rm{\det }}(-J)}\doteq \frac{n(J,E,H)}{{\rm{\det }}(-J)},$$

where det(−*J*) > 0, in view of stability. Each entry *K*_*ij*_ of the influence matrix can be computed by evaluating the sign of *n*(*J*, *E*, *H*) in (7) when *E* = *E*_*j*_ and *H* = *H*_*i*_ have a single non-zero entry (the *j*-th and the *i*-th, respectively) equal to one.

To evaluate the *qualitative* (parameter-free) input-output influence, ref. 27 proposes a vertex algorithm (applicable to any system that admits a so-called *BDC*-decomposition^27, 34, 35^) to assess if increasing the input always results in an *increase* in the output steady-state value, if it always results in a *decrease*, if the steady-state output is *unchanged*, or if the behaviour is *parameter-dependent*. Along the same lines, we can apply a vertex algorithm to uncertain community matrices where each entry lies within a known interval: ${J}_{ij}\in [{J}_{ij}^{-},{J}_{ij}^{+}]$ (e.g., ${J}_{ij}\in [{J}_{ij}^{\ast }-{\varepsilon }_{ij},{J}_{ij}^{\ast }+{\varepsilon }_{ij}]$). In fact, multiaffinity of *n*(*J*, *E*, *H*) with respect to the entries of *J* guarantees the following result.

**Theorem 4** *Denote by J* ^(*v*)^, $v=1,\ldots ,{2}^{{n}^{2}}$, *the community matrices corresponding to all of the possible choices of the entries with* ${J}_{ij}\in \{{J}_{ij}^{-},{J}_{ij}^{+}\}$. *Then*,

- *n*(*J*, *E*, *H*) = 0 *for all matrices J with* ${J}_{ij}\in [{J}_{ij}^{-},{J}_{ij}^{+}]$ *iff n*(*J* ^(*v*)^, *E*, *H*) = 0 *for all v*,
- *n*(*J*, *E*, *H*) > 0 *for all matrices J with* ${J}_{ij}\in [{J}_{ij}^{-},{J}_{ij}^{+}]$ *iff n*(*J* ^(*v*)^, *E*, *H*) > 0 *for all v*,
- *n*(*J*, *E*, *H*) < 0 *for all matrices J with* ${J}_{ij}\in [{J}_{ij}^{-},{J}_{ij}^{+}]$ *iff n*(*J* ^(*v*)^, *E*, *H*) < 0 *for all v*,
- *n*(*J*, *E*, *H*) > 0 *for all matrices J with* ${J}_{ij}\in ({J}_{ij}^{-},{J}_{ij}^{+})$ *iff n*(*J* ^(*v*)^, *E*, *H*) ≥ 0 *for all v and n*(*J* ^(*v*)^, *E*, *H*) > 0 *for some v*,
- *n*(*J*, *E*, *H*) < 0 *for all matrices J with* ${J}_{ij}\in ({J}_{ij}^{-},{J}_{ij}^{+})$ *iff n*(*J* ^(*v*)^, *E*, *H*) ≤ 0 *for all v and n*(*J* ^(*v*)^, *E*, *H*) < 0 *for some v*.

The same algorithm allows us to evaluate also the effect of fixing the abundance of certain species *j* to a new level. Given the unperturbed dynamics $\dot{x}(t)=f(x(t))$ of the community, having *x*_*j*_ suddenly fixed to a new constant level ${\bar{x}}_{j}$ can be seen as adding a constant perturbation, hence $u={\bar{x}}_{j}$. We can then compute the effect on the community based on the formula (7), where now *y* = *x*_*i*_ and *H* = *H*_*i*_, with *i* = 1, …, *n*, *i* ≠ *j*, *J* is the Jacobian matrix of the new system including the densities of all species but species *j*, and vector *E* describes how ${\bar{x}}_{j}$ affects the equations of all the other species. Hence, using the same vertex algorithm proposed for computing the influence matrix, we can compute a vector *v* of dimension *n* − 1 whose entry *v*_*i*_ describes how fixing the abundance *x*_*j*_ to the new level ${\bar{x}}_{j}$ affects the new resulting equilibrium value of species *i* (*i* = 1, …, *n*, *i* ≠ *j*).

Moreover, the proposed vertex algorithm can be used to determine the maximum and the minimum value of the response to a press perturbation, given the known uncertainty bounds (even when the response turns out to have an undetermined sign). Indeed, it is enough to compute the function *F*(*J*, *E*, *H*) = *n*(*J*, *E*, *H*)/det(−*J*), instead of just *n*(*J*, *E*, *H*), for all the vertices of the hyper-rectangle in the parameter space. Then, since the function *F*(*J*, *E*, *H*) is multi-affine in the parameters^58, 59^, both the maximum and the minimum value are achieved on some vertex.

## Electronic supplementary material

Supplementary Material
