# Supplementary material for: Qualitative and quantitative responses to press perturbations in ecological networks
Source: Sci Rep. 2017 Sep 12;7:11378. doi: 10.1038/s41598-017-11221-0 (PMC5596000; doi:10.1038/s41598-017-11221-0)
Supplement: Supplementary file 1 — Supplementary Material [file 41598_2017_11221_MOESM1_ESM.pdf]

Qualitative and quantitative responses to press perturbations in ecological networks
—
Supplementary Information

Giulia Giordano[1] and Claudio Altafini[2]

[1] *Department of Automatic Control and LCCC Linnaeus Center, Lund University, SE-223 63, Lund, Sweden.*
Email: `giulia.giordano@control.lth.se`
[2] *Division of Automatic Control, Dept. of Electrical Engineering, Linköping University, SE-58183, Sweden.*
Email: `claudio.altafini@liu.se`

Contents

1 Introduction **2**

2 Responses to press perturbations are input-output steady-state influences **3**
2.1 Ecological networks, press perturbations and influence matrix 3
2.2 Steady-state input-output influences and the influence matrix 4

3 Qualitative responses to press perturbations **7**
3.1 Mutualistic systems 7
3.2 Monotone systems 7

4 Semi-qualitative responses to press perturbations **8**
4.1 Systems with a strongly connected mutualistic backbone 9
4.2 Systems with a strongly monotone backbone 10

5 Quantitative responses to press perturbations **10**
5.1 Eventually nonnegative matrices 11
5.2 Other cases: eventually positive and eventually exponentially positive 11

6 When the community matrix is uncertain: qualitative influence matrix computation **12**

7 Examples of ecological networks **14**
7.1 Plankton-bacteria-protozoa community 14
7.2 Shallow lake community 16

1 Introduction

Theoretical ecology aims at providing both a qualitative and a quantitative understanding of the dynamics of communities and food webs. As a common way to gain insight into this dynamics, ecologists carry out *press perturbation* experiments, where they alter the density of a species and observe how the density of other species in the community changes when the new equilibrium is reached [2]. Responses to press perturbations are the result of both direct and indirect effects [29, 32, 35, 42], while the community matrix (which is the Jacobian matrix of the system of growth equations evaluated at an equilibrium [25], analysed qualitatively in terms of signed entries, graphs and loops since [26, 27]) only describes direct interactions among species in a community near equilibrium. However, responses to press perturbations are difficult to interpret and counterintuitive when indirect effects are not properly taken into account.

In fact, when species i and j are dynamically coupled through intermediary species, a j-species press affects species i through a complex network of direct and indirect interactions. If the perturbation is small enough and the community has a stable equilibrium point, the *net* steady-state effect (combining all direct and indirect effects) is then given by the negative adjoint of the community matrix [8, 9, 10, 11, 12, 26, 27, 28], whose (i, j) entry predicts the overall effect of a j-species press on species i. The negative inverse of the community matrix can be equivalently considered [2, 23, 35, 42], since, under stability assumptions, the inverse and the adjoint of a matrix have the same sign pattern.

The entries of the community matrix are highly uncertain, due to the lack of knowledge about direct species interactions, and the huge uncertainties that affect ecological network models [33, 22] often prevent from predicting even the sign of the variation. We would like to be able to assess whether, after the press perturbation, the population density at the new equilibrium increases or decreases or remains the same, with respect to the previous equilibrium. In particular, the response of species i to a j-species press perturbation is '0' if the ensuing steady-state variation in the amount of i is zero, or '$+1$' if the sign of the variation is concordant and '-1' if it is discordant with the sign of the press, regardless of the system parameters. Conversely, adopting a qualitative (or structural, parameter-free) approach, the response is indeterminate ('?') if the sign of the steady-state variation depends on the chosen parameters, see Fig. 1(b) of the Main Paper. In this Supplementary Information file,

- following [18, 19] we discuss how the response to a press perturbation can be mathematically seen as a *steady-state input-output influence*, we introduce the *steady-state influence matrix* of a stable system, whose (i, j) entry represents the sign of the steady-state variation of the ith system variable due to a persistent step input applied to the jth system equation (namely, the sign of the shift in the equilibrium of species i after a j-species press) and we point out that the steady-state influence matrix is the sign pattern of the adjoint of the negative of the community matrix of the system (**Section 2**);
- we point out that, for some relevant classes of ecological networks (including all mutualistic and monotone networks, regardless of their topological structure), responses to press perturbations can be evaluated based on a *qualitative approach* that exclusively relies on the knowledge of the sign pattern of the community matrix or, equivalently, of the species direct-interaction graph (**Section 3**);
- for other classes of networks, we propose *semi-qualitative* (**Section 4**) or *quantitative* (**Section 5**) *approaches* that provide useful information on the sign of press perturbation responses;
- we discuss a *computational test* to assess whether the sign of a press perturbation response is always the same, even in the presence of *parametric uncertainties*, by exploiting the multi-affine structure of the problem (**Section 6**);

- we analyse in detail some of the examples discussed in the Main Paper (**Section 7**).

2 Responses to press perturbations are input-output steady-state influences

2.1 Ecological networks, press perturbations and influence matrix

An ecological network that includes all biological interactions occurring in an ecosystem can be seen as a compendium of elementary interactions among species. Hence, it can be effectively visualised as a graph, where the nodes represent species and the edges represent interactions among species. Single and pairwise interactions that normally appear in this context are shown in Fig. 1(a) of the Main Paper, along with their graph representation. The signed graph $\mathcal{G}(S)$, which includes all the elementary interactions occurring among the n species involved, represents the whole ecological network: matrix $S \in \mathbb{R}^{n \times n}$ has entries $[S]_{ij}$ equal to $+1$ if a positive edge goes from j to i, -1 if a negative edge goes from j to i, and 0 otherwise.

The overall nonlinear dynamical system that represents the evolution of an n-species community is

$$\dot{x}(t) = f(x(t)), \tag{1}$$

where the ith component of vector

$$x(t) = [x_1(t) \dots x_n(t)]^\top$$

represents the population density of species i and the ith component of the vector function

$$f(x(t)) = [f_1(x(t)) \dots f_n(x(t))]^\top$$

is the corresponding overall growth rate, which depends on (some or all of) the species densities.

Assumption 1 *The system admits an asymptotically stable equilibrium point $\bar{x}$, such that*

$$f(\bar{x}) = 0.$$

The *community matrix*

$$J = \left. \frac{\partial f(x)}{\partial x} \right|_{x=\bar{x}} \tag{2}$$

is the Jacobian matrix of system (1) evaluated at the equilibrium $\bar{x}$. The entry $[J]_{ij}$ of the community matrix expresses the direct effect of species j on the growth rate of species i.

If we consider the signs of J, we can say that each species has a positive/negative direct influence, or no direct influence, on each of the other species. This is visually represented in the associated graph by a positive/negative edge, or no edge, between the two corresponding nodes. Therefore, there is an equivalence between the overall network graph $\mathcal{G}(S)$ and the sign pattern of the community matrix J: denoting by $\mathrm{sgn}(\cdot)$ the elementwise sign function ($\mathrm{sgn}(k) = +1$ if $k > 0$, $\mathrm{sgn}(k) = -1$ if $k < 0$, $\mathrm{sgn}(k) = 0$ if $k = 0$), we have $\mathrm{sgn}(J) = S$.

Assumption 2 *Each species has a negative self-loop (*e.g.*, due to a density-dependent growth rate).*

The above assumption is needed to guarantee stability of the dynamical system (1) at $\bar{x}$.

Remark 1 *Since we work under asymptotic stability assumptions, we have that* $\det(-J) > 0$ *(this is a necessary condition, see e.g. [17]), and therefore matrix* J *is invertible.*

While J includes direct effects only, the net steady-state influence, combining all direct and indirect effects, is given by the negative adjoint matrix of the community matrix [8, 9, 10, 11, 12, 26, 27, 28]:

$$M = \mathsf{adj}(-J).$$

Entry $[M]_{ij}$ predicts the response of species i to a press perturbation on species j: if the density of species j is experimentally altered and held at a higher level, then, at the new equilibrium, the density of species i will be higher if $[M]_{ij} > 0$, lower if $[M]_{ij} < 0$ and unchanged if $[M]_{ij} = 0$. Since

$$\mathsf{adj}(-J) = (-J)^{-1} \det(-J),$$

and $\det(-J) > 0$ in view of the stability assumption, we can equivalently consider the sign pattern of $-J^{-1}$ [2, 35, 42]. The *influence matrix* is indeed the sign pattern matrix

$$K = \mathrm{sgn}(-J^{-1}) = \mathrm{sgn}[\mathsf{adj}(-J)], \tag{3}$$

expressing the qualitative effect of all species presses on all other species.

2.2 Steady-state input-output influences and the influence matrix

Here, for the sake of completeness, we report material from [19] to show how responses to press perturbations can be seen, more in general, as steady-state input-output influences. This provides a useful insight that helps assess them, even in case of uncertainties (this aspect will be addressed in **Section 6**).

Given a generic nonlinear system, we can see a certain variable of the system as the system *output* and another relevant variable or parameter of the system as the system *input*. Then, the *steady-state input-output influence* is the ensuing variation of the steady state of the system output, upon a variation in the system input. Of course, different outputs (chosen as different variables of the system) may respond with a steady-state variation that has the same sign as the input variation, the opposite sign, or is zero. We say that the steady-state input-output influence is *qualitatively signed* if it always has the same sign (positive, negative, or zero), regardless of the choice of parameter values in the system (see [19]).

To assess the steady-state input-output influence in the nonlinear system

$$\dot{x}(t) = f(x(t), u(t)), \tag{4}$$
$$y(t) = g(x(t)), \tag{5}$$

where $f(\cdot, \cdot)$ and $g(\cdot)$ are continuously differentiable, $x \in \mathbb{R}^n$, $u \in \mathbb{R}$ is an input and $y \in \mathbb{R}$ is an output, we make the following assumptions.

Assumption 3 *There exists an asymptotically stable equilibrium point* $\bar{x}$*, corresponding to* $\bar{u}$*, such that* $f(\bar{x}, \bar{u}) = 0$*.*

Then, both the state asymptotic value $\bar{x}(u)$ and the output asymptotic value $\bar{y}(u) = g(\bar{x})$ are functions of u.

Assumption 4 *The considered input perturbation* u *is small enough to ensure that the stability of* $\bar{x}(u)$ *is preserved.*

Note that the eigenvalues of the Jacobian matrix (based on which the local linearised stability of the equilibrium point is assessed) are continuously dependent on the matrix entries, which are in turn continuous functions of u.

Then, the implicit function theorem provides an analytic expression for the derivative of the steady-state input-output map that relates y to u in system (4)-(5):

$$\frac{\partial \bar{y}}{\partial \bar{u}} = \left.\frac{\partial g}{\partial x}\right|_{\bar{x}} \left(-\left.\frac{\partial f}{\partial x}\right|_{(\bar{x},\bar{u})} \right)^{-1} \left.\frac{\partial f}{\partial u}\right|_{(\bar{x},\bar{u})}. \tag{6}$$

Consider the linear approximation of the nonlinear system in a neighbourhood of the equilibrium $\bar{x}$. Then, denoting by $z(t) = x(t) - \bar{x}$, $v(t) = u(t) - \bar{u}$, $w(t) = y(t) - \bar{y}$, the linearised system is

$$\begin{aligned} \dot{z}(t) &= Jz(t) + Ev(t), \\ w(t) &= Hz(t), \end{aligned}$$

where $[J]_{ij} = \left.\frac{\partial f_i}{\partial x_j}\right|_{(\bar{x},\bar{u})}$, $[E]_i = \left.\frac{\partial f_i}{\partial u}\right|_{(\bar{x},\bar{u})}$ and $[H]_j = \left.\frac{dg}{dx_j}\right|_{\bar{x}}$. J is the community matrix, while E and H are a column and a row vector representing, respectively, how the input acts on the system state and how the output depends on the system state in the linearised system.

Then, the input-output influence can be expressed as [19]

$$\frac{\partial \bar{y}}{\partial \bar{u}} = H(-J)^{-1}E = \frac{n(0)}{d(0)}, \tag{7}$$

where

$$d(0) = \det(-J)$$

is always positive, in view of stability, and

$$n(0) = \det \begin{bmatrix} -J & -E \\ H & 0 \end{bmatrix}. \tag{8}$$

Remark 2 *From a control-theoretic perspective, $n(0)$ and $d(0)$ are the numerator and the denominator of the system* transfer function $F(s) = n(s)/d(s) = H(sI - J)^{-1}E$*, computed at $s = 0$ (for further information concerning Laplace transform and transfer functions, see for instance [20]).*

The above expression can be used to evaluate input-output influences for a given choice of the system parameters. To evaluate the *qualitative* (parameter-free) input-output influence, [19] proposes an efficient vertex algorithm. Such an algorithm is applicable to all systems that admit the so-called BDC-decomposition (see [5, 6, 18, 19]); remarkably, *this broad class includes the class of systems with a sign-definite Jacobian matrix.*

As discussed in [19], a qualitative influence is identified whenever, upon a perturbation due to a constant input, the ensuing variation of the steady-state output value has the same sign as the input (positive influence), the opposite sign (negative influence), or is zero (perfect adaptation),[1] for any feasible choice of the model parameters; otherwise, the influence is indeterminate. This is visually represented in Fig. 1(b)

[1] A variable is adaptive if, when a persistent input is applied, after a transient it reverts to its pre-perturbation value. Adaptation is perfect if the pre-perturbation value is exactly recovered at steady state [13, 38, 41].

of the Main Paper. Consistently, the algorithm in [19] yields a "$+1$" if increasing the input structurally results in an *increase* in the steady-state value of the considered variable, a "-1" if it structurally results in a *decrease*, a "0" if the steady-state of the considered variable is *unchanged*, and a "?" if the behaviour is *parameter-dependent*.

When a persistent additive input is applied to a single state equation and a single state variable is taken as the system output, the results for all the possible input-output pair combinations can be visualised in the *influence matrix*, whose (i, j) entry expresses the sign of the overall steady-state influence on the ith system variable of an external persistent additive input applied to the dynamic equation of the jth system variable [19]. Consider a system (4)–(5) of the form

$$\begin{aligned} \dot{x}(t) &= f(x(t)) + Eu(t), \qquad (9) \\ y(t) &= Hx(t), \qquad (10) \end{aligned}$$

with community matrix

$$J = \left.\frac{\partial f(x)}{\partial x}\right|_{x=\bar{x}},$$

where $\bar{x}$ is an asymptotically stable equilibrium, and take vectors $E = E_j$ and $H = H_i$ with a single non-zero entry equal to one

$$E_j = [0 \ \dots \ 0 \ \underbrace{1}_{\text{position } j} \ 0 \ \dots \ 0]^\top, \qquad H_i = [0 \ \dots \ 0 \ \underbrace{1}_{\text{position } i} \ 0 \ \dots \ 0].$$

Each entry $[K]_{ij}$ of the *qualitative influence matrix* $K \in \mathbb{R}^{n \times n}$ (which can be evaluated by the vertex algorithm in [19] for any system that admits a BDC-decomposition) can be:

- '$+1$' if the influence is positive for any choice of the parameters;
- '0' if there is perfect adaptation for any choice of the parameters;
- '-1' if the influence is negative for any choice of the parameters;
- '?' if the influence can have a different sign depending on the chosen parameters.

Note that, as we have mentioned earlier, the influence matrix K corresponds to the sign pattern of the adjoint matrix of the negative of the community matrix J, adj$(-J)$. Since $\det(-J)$ is positive in view of the stability assumption [17], J is invertible,

$$(-J)^{-1} = \frac{1}{\det(-J)} \text{adj}(-J),$$

and the influence matrix has the same sign pattern as $-J^{-1}$:

$$\text{sgn}(K) = \text{sgn}[\text{adj}(-J)] = \text{sgn}[(-J)^{-1}]. \qquad (11)$$

The so-called *structural (i.e., qualitative) influence matrix*, which expresses the overall direct and indirect influences, at steady-state, among the state variables of a dynamical network, regardless of the chosen parameter values, can then be computed by means of the vertex algorithm proposed in [19] for all systems admitting the BDC-decomposition described in [5, 6, 18, 19].

3 Qualitative responses to press perturbations

For particular classes of systems, the signed influence matrix can have special properties that help us evaluate it without any quantitative knowledge about the system. For instance, as pointed out in [18, 19] based on [15], the influence matrix associated with a system whose matrix J is Metzler has exclusively '$+1$' or '0' entries; it has exclusively '$+1$' entries if, besides being Metzler, matrix J is irreducible. This result can be generalised to the class of monotone systems, based on classical results [36, 37, 39]. Indeed, the community matrix of any monotone system becomes Metzler after a so-called "gauge transformation" [14], i.e., a diagonal similarity transformation with diagonal elements ± 1.

3.1 Mutualistic systems

Given matrix $A \in \mathbb{R}^{n\times n}$, $\mathcal{G}(A)$ denotes the digraph with adjacency matrix A, while $Q[A]$ is the *qualitative class* of all matrices having the same sign pattern as A. In particular, $Q[A]$ always contains a signature matrix $S = \mathrm{sgn}(A)$ whose entries are in $\{0, -1, +1\}$. Clearly, $\mathcal{G}(A)$, $\mathcal{G}(S)$ and $\mathcal{G}(B)$ $\forall\, B \in Q[A]$ all have the same (signed) graph, but possibly different numerical weights.

A matrix A is said *irreducible* if no permutation matrix P exists such that

$$P^\top AP = \begin{bmatrix} A_1 & 0 \\ A_2 & A_3 \end{bmatrix}$$

with A_1 and A_3 square matrices; equivalently, $\mathcal{G}(A)$ is strongly connected. Clearly, if A is irreducible, any matrix $B \in Q[A]$ is irreducible, since $\mathcal{G}(A)$ and $\mathcal{G}(B)$ have the same topology.

A mutualistic network has a community matrix J which is Metzler, i.e., the species-species interactions are all mutually beneficial or commensal (see Fig. 1 (a) of the Main Paper) and only self-loops are negative. For Metzler community matrices we have the following.

Theorem 1 *(See [15, 19]) If the community matrix J in* (2) *associated with system* (1) *is stable and Metzler, then $K_{ij} \in \{0, +1\}$ for all $i, j \in \{1, \dots, n\}$. Moreover, if matrix J is also irreducible, then $K_{ij} = +1$ for all $i, j \in \{1, \dots, n\}$.*

A direct consequence of Theorem 1 is the following.

Theorem 2 *Given S mutualistic with $S_{ii} < 0$ for all $i \in \{1, \dots, n\}$, any stable $J \in Q[S]$ has influence matrix K such that $K_{ij} \in \{0, +1\}$ for all $i, j \in \{1, \dots, n\}$. If in addition $\mathcal{G}(S)$ is strongly connected, then $K_{ij} = +1$ for all $i, j \in \{1, \dots, n\}$.*

It is worth stressing that the converse of Theorems 1 and 2 is not true. As discussed in [18, 19], there are systems whose community matrix is not Metzler, and which nonetheless yield a fully positive influence matrix. More on this below.

3.2 Monotone systems

Denote by $x(t)$ the solution of (1) at time t with initial condition $x(0)$. Consider a diagonal matrix $\Sigma = \mathrm{diag}(\sigma)$ with diagonal entries $\sigma = (\sigma_1, \dots, \sigma_n)$, $\sigma_i \in \{\pm 1\}$, which we call gauge matrix [14]. Vector σ identifies a partial order for the n axes of $\mathbb{R}^n$, which can be the "natural" one when all $\sigma_i = +1$, or the opposite when all $\sigma_i = -1$, or any mixed sign combination. The system in (1) is said *monotone* with

respect to the partial order σ if, for all initial conditions $x_1(0)$, $x_2(0)$ such that $\Sigma x_1(0) \leqslant \Sigma x_2(0)$, it is $\Sigma x_1(t) \leqslant \Sigma x_2(t)\, \forall\, t \geqslant 0$ [36, 37, 39]. The ordering is strict if, in addition, strict inequality holds for at least one of the coordinates of x_1, x_2, but not necessarily for all. The system in (1) is said *strongly monotone* with respect to the partial order σ if, for all initial conditions $x_1(0)$, $x_2(0)$ such that $\Sigma x_1(0) \leq \Sigma x_2(0)$, $x_1(0) \neq x_2(0)$, it is $\Sigma x_1(t) < \Sigma x_2(t)\ \forall\, t > 0$. Monotonicity of a system can be checked in terms of its Jacobian $J(x) = \frac{\partial f(x)}{\partial x}$ based on the Kamke condition [36, Lemma 2.1]: the system in (1) is monotone w.r.t. the order σ if and only if

$$\sigma_i \sigma_j J_{ij}(x) \geqslant 0 \qquad \forall\, x \in \mathbb{R}^n, \qquad \forall\, i, j = 1, \dots, n \quad i \neq j, \tag{12}$$

or, in matrix form, $\Sigma J(x) \Sigma$ is Metzler $\forall\, x \in \mathbb{R}^n$. This condition implies that $J(x)$ must have the same signature $S = \mathrm{sgn}(J(x))$ everywhere, hence it can be stated equivalently in terms of S as

$$\sigma_i \sigma_j S_{ij} \geqslant 0 \qquad \forall\, i, j = 1, \dots, n \quad i \neq j. \tag{13}$$

The condition in (13) admits a graph-theoretical reformulation. The system in (1) is monotone with respect to some order if and only if all directed cycles of length > 1 of the signed digraph $\mathcal{G}(S)$ (or $\mathcal{G}(J)$) have positive sign.

Monotonicity, combined with irreducibility of $J(x)$ at all x, implies strong monotonicity of (1).

The following generalisation of Theorems 1 and 2 hold, based on well-known results on monotone systems [36, 37, 39].

Theorem 3 *If system* (1) *is monotone and, given a gauge transformation* Σ*, matrix* $\Sigma J \Sigma$ *is stable and Metzler, then* $K = \Sigma \hat{K} \Sigma$*, where* $\hat{K}$ *is a matrix containing only* 0 *and* $+1$ *terms (only* $+1$ *terms if the system is strongly monotone, namely, matrix* J *is also irreducible).*

Theorem 4 *Given* S *such that* $\Sigma S \Sigma$ *is Metzler for some gauge transformation* Σ *and* $S_{ii} < 0$ *for all* $i \in \{1, \dots, n\}$*, any stable* $J \in Q[S]$ *is such that its influence matrix is* $K = \Sigma \hat{K} \Sigma$*, where* $\hat{K}$ *is a matrix containing only* 0 *and* $+1$ *terms (only* $+1$ *terms if the system is strongly monotone, namely,* $\mathcal{G}(S)$ *is also strongly connected).*

4 Semi-qualitative responses to press perturbations

It is known, see for instance [18, 19], that even community matrices that are not Metzler can give rise to elementwise positive influence matrices, meaning that complete mutualism of the community matrix is not necessary, but just sufficient for the system to yield a nonnegative (and positive in the irreducible case) influence matrix.

However, when $\mathcal{G}(S)$ is not mutualistic, we cannot resort to a purely qualitative approach to determine the signature of the influence matrix K. Only *semi-qualitative* graph-based conditions can be stated to identify community matrices that may yield a positive influence matrix for some choice of the values of the parameters.

Here, we give a sufficient condition for a system to possibly admit a positive influence matrix (for some values of the community matrix entries) that is exclusively based on the sign pattern of $\mathcal{G}(S)$: the qualitative class $Q[S]$ (in which all matrices have the same topology and the same signature of $S = \mathrm{sgn}(J)$) can contain at least one community matrix with a positive influence matrix provided that the subgraph $\mathcal{G}(S^+)$ (formed by taking only the positive edges of $\mathcal{G}(S)$) forms a network-wide strongly connected component. This can be seen as a strongly connected mutualistic "backbone".

4.1 Systems with a strongly connected mutualistic backbone

Given a matrix A, let A^+ be the nonnegative part of A,

$$A^+_{ij} = \begin{cases} A_{ij} & \text{if } A_{ij} \geq 0 \\ 0 & \text{if } A_{ij} < 0 \end{cases}$$

and $\hat{A}$ the following "lifting" of A to $\mathbb{R}^{2n\times 2n}$ (see e.g. [7]):

$$\hat{A} = \begin{bmatrix} 0 & A \\ -A^\top & 0 \end{bmatrix}^+ .$$

A matrix A is *fully indecomposable* if no permutation matrices P_1, P_2 exist such that

$$P_1 A P_2 = \begin{bmatrix} A_1 & 0 \\ A_2 & A_3 \end{bmatrix}$$

where A_1 and A_3 are square matrices. Matrix A is fully indecomposable if and only if, for some permutation matrix P, PA is irreducible and has nonzero diagonal entries (see for instance [4, p. 56]). We have the following theorem by Fiedler and Grone [16].

Theorem 5 *[16] Given a fully indecomposable signature matrix S, the following are equivalent:*

1. *there exists a matrix $B \in Q[S]$ such that $B^{-1} > 0$;*
2. *matrix $\hat{S} = \begin{bmatrix} 0 & S \\ -S^\top & 0 \end{bmatrix}^+$ is irreducible;*
3. *S cannot be expressed in the form $P_1 \begin{bmatrix} S_{11} & S_{12} \\ S_{21} & S_{22} \end{bmatrix} P_2$,*

   where S_{11} need not be square, P_1 and P_2 are permutation matrices, $S_{12} \geqslant 0$ and $S_{21} \leqslant 0$, with at least one of these two blocks being nonvoid.

Based on this result, we can derive qualitative sufficient conditions that allow a matrix with a given sign structure to have a negative inverse with positive entries. In particular, we can prove the following.

Theorem 6 *Given an irreducible matrix S, with $S_{ii} = -1 \; \forall\, i = 1, \dots, n$, if matrix S^+ is irreducible, then there exists a matrix $J \in Q[S]$ such that $-J^{-1} > 0$.*

Proof. Consider matrix $-S$: since $-S$ is irreducible and $-S_{ii} > 0 \; \forall\, i$, then $-S$ is fully indecomposable, as required by Theorem 5. Consider then the corresponding lifting

$$\hat{S}_{neg} = \begin{bmatrix} 0 & -S \\ S^\top & 0 \end{bmatrix}^+ .$$

By construction, its upper right block has all nonzero diagonal entries, hence in the bipartite graph $\mathcal{G}(\hat{S}_{neg})$ there exists a direct edge from each node $n+i$ to node i, with $i \in \{1, \dots, n\}$. If S^+ is irreducible, then $(S^\top)^+$ is irreducible as well and there exists a path in $\mathcal{G}(\hat{S}_{neg})$ between each pair of nodes j and $n+i$,

with $i, j \in \{1, \ldots, n\}$. Hence, for any pair $i, j \in \{1, \ldots, n\}$, there exists a path $n + j \to j \to n + i \to i$, which means that the graph $\mathcal{G}(\hat{S}_{neg})$ is strongly connected, thus $\hat{S}_{neg}$ is irreducible.

Therefore, in view of Theorem 5, for some $B \in Q[-S]$ it must be $B^{-1} > 0$. If we choose $J = -B$, then $J \in Q[S]$ and $-J^{-1} = B^{-1} > 0$. ■

The converse of Theorem 6 is not true, as the following example shows.

Example 1 Consider the irreducible signature matrix

$$S = \begin{bmatrix} -1 & -1 & -1 & 1 \\ -1 & -1 & 1 & -1 \\ -1 & 1 & -1 & -1 \\ 1 & 0 & -1 & -1 \end{bmatrix}.$$

The corresponding S^+ is clearly reducible, but matrix

$$\hat{S} = \left[\begin{array}{cccc|cccc} 0 & 0 & 0 & 0 & 0 & 0 & 0 & 1 \\ 0 & 0 & 0 & 0 & 0 & 0 & 1 & 0 \\ 0 & 0 & 0 & 0 & 0 & 1 & 0 & 0 \\ 0 & 0 & 0 & 0 & 1 & 0 & 0 & 0 \\ \hline 1 & 1 & 1 & 0 & 0 & 0 & 0 & 0 \\ 1 & 1 & 0 & 0 & 0 & 0 & 0 & 0 \\ 1 & 0 & 1 & 1 & 0 & 0 & 0 & 0 \\ 0 & 1 & 1 & 1 & 0 & 0 & 0 & 0 \end{array}\right]$$

is irreducible. Hence, Theorem 6 cannot be applied, while Theorem 5 still holds.

4.2 Systems with a strongly monotone backbone

Theorem 6 can be extended to systems that have a strongly monotone backbone, i.e., such that $\mathcal{G}\big((\Sigma S \Sigma)^+\big)$ is strongly connected, where Σ is a gauge transformation matrix.

Theorem 7 *Given an irreducible matrix S, with $S_{ii} = -1 \; \forall\, i = 1, \ldots, n$, if there exists a gauge transformation Σ such that the matrix $(\Sigma S \Sigma)^+$ is irreducible, then there exists a matrix $J \in Q[S]$ such that $-(\Sigma J \Sigma)^{-1} = \Sigma(-J^{-1})\Sigma > 0$.*

5 Quantitative responses to press perturbations

In this section, we describe a class of matrices J that admit a positive influence matrix $K = \text{sign}(-J^{-1})$, although they are not associated with mutualistic systems. These matrices are related to eventually nonnegative matrices [30, 31]: a matrix is eventually nonnegative if it becomes elementwise nonnegative after a certain power (more rigorous definition below). Then, eventually nonnegative matrices with a proper diagonal shift lead to community matrices having a positive influence matrix. In fact, if we consider an irreducible and eventually nonnegative matrix B, then there exists an interval $(\rho(B), \beta)$ of the real line (where $\rho(B)$ is the spectral radius of B) such that for all $\alpha \in (\rho(B), \beta)$, matrix $J = B - \alpha I$ is stable and such that $(-J)^{-1} > 0$, implying that $K > 0$. This can be verified directly using the vertex algorithm

described in Theorem 11, with uncertainty only on the diagonal parameters: $J_{ii} \in [\rho(B) - \beta,\, 0]$. Notice that B eventually nonnegative implies that also J is eventually nonnegative, but with eigenvalues of different real part (more inside the left half of the complex plane, since $\alpha > 0$). In fact, in $J = B - \alpha I$, the diagonal term αI plays the same role as the diagonal of a Metzler matrix: it guarantees Hurwitz stability of J, which in turn ensures that $\det(-J) > 0$. Since $\alpha > \rho(B)$, stability holds regardless of the values on the diagonal of B.

5.1 Eventually nonnegative matrices

A matrix $M \in \mathbb{R}^{n\times n}$ is *eventually nonnegative* if $\exists\, p_0 \in \mathbb{N}$ such that, $\forall\, p \geq p_0$, $M^p \geq 0$ elementwise; equivalently, its spectral radius

$$\rho(M) = \max_{\lambda_i \in \sigma(M)} |\lambda_i|$$

is a real, positive eigenvalue of M, called the Perron-Frobenius eigenvalue, and the corresponding left and right eigenvectors are elementwise nonnegative. Denote by $\mathrm{index}_\lambda(M)$ the multiplicity of the eigenvalue λ of M as a root of the minimal polynomial (*i.e.*, the dimension of the largest Jordan block associated with λ). Then we have the following result, adapted from [24, Theorem 4.2].

Theorem 8 *Consider $J = B - \alpha I$, where $B \in \mathbb{R}^{n\times n}$ is irreducible and eventually nonnegative, with $\mathrm{index}_0(B) \leqslant 1$. Then, $\exists\, \beta > \rho(B)$ such that $\forall\, \alpha \in (\rho(B),\, \beta)$, $-J = \alpha I - B$ has a positive inverse.*

More generally, if $\exists\, \alpha$ such that $J + \alpha I = B$ is eventually nonnegative and satisfies Theorem 8, then the influence matrix derived from J is positive: $(-J)^{-1} > 0$, hence $K > 0$ elementwise.

Remark 3 *The condition $\mathrm{index}_0(B) \leqslant 1$ is generically verified if B is irreducible, at least when the coefficients of B are drawn randomly. All eigenvalues, including the 0 eigenvalue, are generically simple in this case.*

Note that the converse of Theorem 8 is not true.

5.2 Other cases: eventually positive and eventually exponentially positive

Other, similar, cases are described in [34]. For instance, if we consider the closely related class of eventually positive matrices, then it is possible to obtain qualitative conditions in the spirit of those discussed in the previous sections, namely conditions on the sign pattern that forbid a certain qualitative class of matrices to have a representative that is eventually positive. A matrix $M \in \mathbb{R}^{n\times n}$ is *eventually positive* if $\exists\, p_0 \in \mathbb{N}$ such that, $\forall\, p \geq p_0$, $M^p > 0$ elementwise; equivalently, its Perron-Frobenious eigenvalue $\rho(M)$ is real, positive, and the corresponding left and right eigenvectors are elementwise positive. A first necessary condition for a qualitative class $Q[S]$ to contain an eventually positive matrix is that S is irreducible [3]. The following theorem is also from [3].

Theorem 9 *([3], Thm. 5.2) Consider an irreducible signature matrix S. If S has the block sign pattern*

$$\begin{bmatrix} S_{11} & S_{12} \\ S_{21} & S_{22} \end{bmatrix}$$

where S_{11} and S_{22} are square matrices and $S_{12} = S_{12}^{+}$, $-S_{21} = (-S_{21})^{+}$, then $\nexists\, B \in Q[S]$ such that B is eventually positive.

A matrix M is *eventually exponentially positive* if $\exists\, t_0 \in \mathbb{R}$ such that, $\forall\, t \geq t_0$, $e^{Mt} > 0$ elementwise; equivalently, the matrix enjoys the Perron-Frobenious property in terms of spectral abscissa: its dominant eigenvalue $\lambda^* = \arg\max_{\lambda_i \in \sigma(M)} \Re(\lambda_i)$ (namely, that having the *maximum real part*) is real and the corresponding left and right eigenvectors are elementwise positive.

The following result, adapted from [30, Theorem 2.2], [1, Lemma 2], links eventual positivity with eventual exponential positivity. It allows to better understand the role of α in $J = B - \alpha I$.

Theorem 10 *A matrix $A \in \mathbb{R}^{n\times n}$ is eventually exponentially positive if and only if $A + \alpha I$ is eventually positive for some $\alpha \geq 0$.*

Remark 4 *We can provide a graph-theoretical interpretation of Theorems 8 and 10. If M is the adjacency matrix of a directed graph (so that $|M_{ij}| = 1$ if an edge connects nodes i and j, $M_{ij} = 0$ otherwise), then its power M^k is such that M^k_{ij} is equal to the number of paths of length k that connect nodes i and j. Then, let B be an eventually nonnegative or eventually positive matrix and the adjacency matrix of a weighted directed graph (we can set $B_{ii} = 0$, since self-loops are not relevant). The (i, j) entry of matrix B^k,*

$$B^k_{ij} = \sum_{h_1,h_2,\ldots,h_{k-1}} B_{i,h_1} B_{h_1,h_2} \ldots B_{h_{k-1},j},$$

is the sum of all possible edge products (where each edge is weighted by the corresponding entry of B) corresponding to all possible paths of length k in the graph. Hence, the sum of all possible paths of length k becomes positive for large k. When B is eventually positive, in the expression of the exponential matrix of $J = B - \alpha I$, it is

$$e^{(B-\alpha I)t} = e^{Bt} e^{-\alpha It} = \sum_{k=0}^{\infty} \frac{B^k t^k}{k!} e^{-\alpha It},$$

where $e^{-\alpha It}$ is a diagonal matrix with positive diagonal entries and, in the infinite sum, the terms corresponding to powers B^k with $k > k_o$ provide a positive contribution, because the sum of all possible paths of length $k > k_o$ is positive in the graph.

6 When the community matrix is uncertain: qualitative influence matrix computation

Following the approach in Section 2 and in [19], given $x \in \mathbb{R}^n$, we consider the nonlinear system

$$\dot{x}(t) = f(x(t)) + Eu(t), \quad y(t) = Hx(t), \tag{14}$$

where $f(\cdot)$ is continuously differentiable, $u \in \mathbb{R}$ is an input, $y \in \mathbb{R}$ is an output, and we assume that there exists an asymptotically stable equilibrium point $\bar{x}$. Then, both the state asymptotic value $\bar{x}(u)$ and the output asymptotic value $\bar{y}(u) = H\bar{x}$ are functions of u. The *steady-state input-output influence* [19] is the ensuing variation of the steady state of the system output y, upon a variation in the input u (a relevant variable or parameter). We assume that the considered input perturbation is small enough to ensure that the stability of $\bar{x}(u)$ is preserved (being the eigenvalues of the Jacobian matrix continuously dependent on the entries, which are in turn continuous functions of u). Of course, different variables of interest for the system may respond with a steady-state variation that has the same sign as the input

variation, the opposite sign, or is zero. The steady-state input-output influence is *qualitatively signed* if it always has the same sign (positive, negative, or zero), regardless of the choice of parameter values [19]. Denoting by J the community matrix, in view of the implicit function theorem, the input-output influence (or sensitivity) can be expressed as [19]

$$\frac{\partial \bar{y}}{\partial \bar{u}} = H(-J)^{-1}E = \frac{\det \begin{bmatrix} -J & -E \\ H & 0 \end{bmatrix}}{\det(-J)} \doteq \frac{n(J,E,H)}{\det(-J)}, \tag{15}$$

where $\det(-J) > 0$, in view of stability. Each entry K_{ij} of the influence matrix can be computed by evaluating the sign of $n(J,E,H)$ in (15) when $E = E_j$ and $H = H_i$ have a single non-zero entry (the $j$the and the ith, respectively) equal to one.

To evaluate the *qualitative* (parameter-free) input-output influence, [19] proposes a vertex algorithm (applicable to any system that admits a BDC-decomposition [5, 6, 19]) to assess if increasing the input always results in an *increase* in the output steady-state value, if it always results in a *decrease*, if the steady-state output is *unchanged*, **regardless of the choice of parameter values**, or if the behaviour is *parameter-dependent*. Along the same lines, we can apply a vertex algorithm to uncertain community matrices where each entry **belongs to a known (possibly bounded) interval**: $J_{ij} \in [J_{ij}^-, J_{ij}^+]$ (e.g., $J_{ij} \in [J_{ij}^* - \epsilon_{ij}, J_{ij}^* + \epsilon_{ij}]$). For instance, we might have that the (i,j) entry has a nominal value J_{ij}^* that is affected by an uncertainty of amplitude ϵ_{ij}, hence $J_{ij} \in [J_{ij}^* - \epsilon_{ij}, J_{ij}^* + \epsilon_{ij}]$.

In fact, also in the case of uncertain parameters belonging to given intervals, multiaffinity of $n(J,E,H)$ with respect to the entries of J guarantees the following result.

Theorem 11 *Denote by $J^{(v)}$, $v = 1, \ldots, 2^{n^2}$, the community matrices corresponding to all of the possible choices of the entries with $J_{ij} \in \{J_{ij}^-, J_{ij}^+\}$. Then,*

1. *$n(J,E,H) = 0$ for all matrices J with $J_{ij} \in [J_{ij}^-, J_{ij}^+]$ iff $n(J^{(v)},E,H) = 0$ for all v,*
2. *$n(J,E,H) > 0$ for all matrices J with $J_{ij} \in [J_{ij}^-, J_{ij}^+]$ iff $n(J^{(v)},E,H) > 0$ for all v,*
3. *$n(J,E,H) < 0$ for all matrices J with $J_{ij} \in [J_{ij}^-, J_{ij}^+]$ iff $n(J^{(v)},E,H) < 0$ for all v,*
4. *$n(J,E,H) > 0$ for all matrices J with $J_{ij} \in (J_{ij}^-, J_{ij}^+)$ iff $n(J^{(v)},E,H) \geq 0$ for all v and $n(J^{(v)},E,H) > 0$ for some v,*
5. *$n(J,E,H) < 0$ for all matrices J with $J_{ij} \in (J_{ij}^-, J_{ij}^+)$ iff $n(J^{(v)},E,H) \leq 0$ for all v and $n(J^{(v)},E,H) < 0$ for some v.*

Proof. Necessity is immediate in view of continuity. Sufficiency can be proved relying on the multiaffinity of $n(J,E,H)$ with respect to the entries of J. In fact, a multiaffine function defined on a hypercube reaches its minimum (and maximum) value on a vertex of the hypercube. We prove sufficiency for the second claim (the others can be proved similarly), by contradiction. Being the function multiaffine, it must be $n(J,E,H) \geq 0$ in the whole hypercube. Assume there is an internal point of the hypercube such that $n(J,E,H) = 0$. Then, consider variations along the direction of $J_{11}^- \leq J_{11} \leq J_{11}^+$. The restricted function is linear and nonnegative. Hence, if it is zero at one point, it must be zero at both the extrema: $n(J_-^{(1)},E,H) = n(J_+^{(1)},E,H) = 0$, where $J_-^{(1)}$ is the matrix where $J_{11} = J_{11}^-$ and $J_+^{(1)}$ is the matrix where $J_{11} = J_{11}^+$. If we fix first $J_{11} = J_{11}^+$ and then $J_{11} = J_{11}^-$, in both cases we can repeat the same argument along the direction of each of the following entries, to conclude that it must be $n(J,E,H) = 0$ for all the vertices of the matrix. However, this is in contradiction with the assumption that $n(J^{(v)},E,H) > 0$ for some v. Hence, it must be $n(J,E,H) > 0$ for all internal points of the hypercube. ■

Remark 5 *The number of community matrices to be tested is 2^q, where q is the number of uncertain nonzero entries. Therefore, $q = n^2$ in the worst case. However, in ecological networks, community matrices are typically sparse, hence we can expect to have $q \ll n^2$.*

The computational effort is paid back by a very strong knowledge. If we get a qualitative answer for an entry, then the response to that press perturbation will have the same sign *for all possible community matrices having their entries in the given intervals*.

7 Examples of ecological networks

7.1 Plankton-bacteria-protozoa community

Consider the ecological network described in [40, 9], whose interaction graph $\mathcal{G}(S)$ is shown in Fig. 3(a) of the Main Paper. The nominal value of the community matrix according to [40], is

$$J = \begin{bmatrix} -1 & 0.6 & 0 & 0 & 0 \\ -0.6 & -1 & 0.6 & 0.1 & 0 \\ 0.6 & -0.6 & -1 & -0.5 & 0.2 \\ 0 & 0 & 0.5 & -1 & -0.2 \\ 0 & 0 & 0 & 0.2 & -1 \end{bmatrix}, \tag{16}$$

which yields the sign pattern matrix

$$S = \begin{bmatrix} -1 & 1 & 0 & 0 & 0 \\ -1 & -1 & 1 & 1 & 0 \\ 1 & -1 & -1 & -1 & 1 \\ 0 & 0 & 1 & -1 & -1 \\ 0 & 0 & 0 & 1 & -1 \end{bmatrix}. \tag{17}$$

The influence matrix corresponding to the nominal community matrix has sign pattern

$$K = \begin{bmatrix} 1 & 1 & 1 & -1 & 1 \\ -1 & 1 & 1 & -1 & 1 \\ 1 & -1 & 1 & -1 & 1 \\ 1 & -1 & 1 & 1 & -1 \\ 1 & -1 & 1 & 1 & 1 \end{bmatrix}. \tag{18}$$

What happens in the case of uncertainties in the community matrix (16)? The vertex algorithm described in the previous section (see Theorem 11) allows us to certify that the sign pattern in (18) is preserved, *no matter how the community matrix entries vary within bounded intervals*, as follows:

$$\begin{bmatrix} -1 \pm 0.15 & 0.6 \pm 0.1 & 0 & 0 & 0 \\ -0.6 \pm 0.1 & -1 \pm 0.15 & 0.6 \pm 0.1 & 0.1 \pm 0.1 & 0 \\ 0.6 \pm 0.1 & -0.6 \pm 0.01 & -1 \pm 0.01 & -0.5 \pm 0.01 & 0.2 \pm 0.1 \\ 0 & 0 & 0.5 \pm 0.1 & -1 \pm 0.1 & -0.2 \pm 0.01 \\ 0 & 0 & 0 & 0.2 \pm 0.01 & -1 \pm 0.1 \end{bmatrix}, \tag{19}$$

where the notation $J_{ij} = \bar{J}_{ij} \pm \delta_{ij}$ means that the entry can take values in the whole interval $J_{ij} \in [\bar{J}_{ij} - \delta_{ij},\, \bar{J}_{ij} + \delta_{ij}]$.

As shown above, the nominal choice of the parameters (16) and the polytope of uncertainty given in (19) lead to the influence matrix (18), which is not fully positive. However, since the graph $\mathcal{G}(S^+)$ (obtained by taking only the positive edges of $\mathcal{G}(S)$) is strongly connected, the community must yield a fully positive influence matrix for some other choice of the parameters having the sign pattern (17), in view of Theorem 6.

An example is given by the choice

$$\tilde{J}_{pos} = \begin{bmatrix} \bar{d}_1 & \bar{d}_2 & 0 & 0 & 0 \\ \bar{d}_3 & \bar{d}_4 & \bar{d}_5 & \bar{d}_6 & 0 \\ \bar{d}_7 & \bar{d}_8 & \bar{d}_9 & \bar{d}_{10} & \bar{d}_{11} \\ 0 & 0 & \bar{d}_{12} & \bar{d}_{13} & \bar{d}_{14} \\ 0 & 0 & 0 & \bar{d}_{15} & \bar{d}_{16} \end{bmatrix} = \begin{bmatrix} -1 & 0.6 & 0 & 0 & 0 \\ -0.6 & -1 & 1 & 0.1 & 0 \\ 0.6 & -0.2 & -1 & -0.1 & 0.4 \\ 0 & 0 & 0.5 & -1 & -0.2 \\ 0 & 0 & 0 & 0.3 & -1 \end{bmatrix}, \tag{20}$$

which is such that $-\tilde{J}_{pos}^{-1} > 0$ elementwise.

Figure 1 shows the outcome of a numerical sampling of the parameter space. In each of the plots, the influence matrix is computed when two of the d_i's vary in the range $d_i \in [\bar{d}_i - 0.1,\, \bar{d}_i + 0.1]$, where $\bar{d}_i$ is the nominal choice of the parameters in (20), marked in the plots by the cyan diamond. Blue points in the parameter space correspond to parameter choices that lead to a fully positive influence matrix, while red points correspond to parameter choices that do not lead to a fully positive influence matrix.

It is worth pointing out that matrix $\tilde{J}_{pos}$ in (20) is eventually exponentially positive.

According to Theorem 10, since $\tilde{J}_{pos}$ is eventually exponentially positive, there must exist a choice of $\alpha \geq 0$ such that $\tilde{J}_{pos} = B - \alpha I$, with B eventually positive. Indeed, for $\alpha = 1.37$, $B = \tilde{J}_{pos} + \alpha I$ is eventually positive, irreducible and $\mathrm{index}_0(B) \leqslant 1$, with $\rho(B) = 0.898$. Hence, in view of Theorem 8, there exists $\beta > \rho(B)$ such that $\forall\, \alpha \in (\rho(B),\, \beta)$, $\alpha I - B$ has a positive inverse. In this case $\beta = 1.421$. Clearly $\alpha = 1.37$ belongs to the interval $(\rho(B),\, \beta) = (0.898,\, 1.421)$.

Another choice of the community matrix entries that gives a fully positive influence matrix is

$$J_{pos} = \begin{bmatrix} \bar{d}_1 & \bar{d}_2 & 0 & 0 & 0 \\ \bar{d}_3 & \bar{d}_4 & \bar{d}_5 & \bar{d}_6 & 0 \\ \bar{d}_7 & \bar{d}_8 & \bar{d}_9 & \bar{d}_{10} & \bar{d}_{11} \\ 0 & 0 & \bar{d}_{12} & \bar{d}_{13} & \bar{d}_{14} \\ 0 & 0 & 0 & \bar{d}_{15} & \bar{d}_{16} \end{bmatrix} = \begin{bmatrix} -0.6 & 0.6 & 0 & 0 & 0 \\ -0.6 & -0.6 & 0.6 & 1 & 0 \\ 1 & -0.8 & -0.6 & -0.6 & 1 \\ 0 & 0 & 0.6 & -0.6 & -1 \\ 0 & 0 & 0 & 0.6 & -0.6 \end{bmatrix}. \tag{21}$$

Also in this case, $-J_{pos}^{-1} > 0$ elementwise. It is possible to show "robustness" of this parameter choice based on the vertex algorithm (Theorem 11). In fact, running the algorithm certifies that the influence matrix remains fully positive, *no matter how the entries d_i vary within the following intervals*:

$$\begin{bmatrix} -0.6 \pm 0.03 & 0.6 \pm 0.02 & 0 & 0 & 0 \\ -0.6 \pm 0.04 & -0.6 \pm 0.04 & 0.6 \pm 0.04 & 1 \pm 0.04 & 0 \\ 1 \pm 0.02 & -0.8 \pm 0.01 & -0.6 \pm 0.01 & -0.6 \pm 0.05 & 1 \pm 0.02 \\ 0 & 0 & 0.6 \pm 0.01 & -0.6 \pm 0.05 & -1 \pm 0.01 \\ 0 & 0 & 0 & 0.6 \pm 0.05 & -0.6 \pm 0.05 \end{bmatrix}.$$

These intervals are fairly small because we want to guarantee positivity *simultaneously* for all variations. Normally, only some of the parameters are uncertain. For instance, Figures 2 and 3 show the outcome of a numerical sampling of the parameter space when only 2 parameters are varied at a time. In each of the plots, the influence matrix is computed when two of the d_i's vary in the range $d_i \in [\bar{d}_i - 0.1,\, \bar{d}_i + 0.1]$ (Figure 2) or $d_i \in [\bar{d}_i - 0.2,\, \bar{d}_i + 0.2]$ (Figure 3), where $\bar{d}_i$ is the nominal choice of the parameters in (21), marked in the plots by the cyan diamond. Blue points in the parameter space correspond to parameter choices that lead to a fully positive influence matrix, while red points correspond to parameter choices that do not lead to a fully positive influence matrix and green points correspond to parameter choices that make the community matrix singular (hence, its inverse cannot be computed). It can be seen that all points included in the hyper-rectangle of parameters that has been successfully tested with the vertex algorithm yield, as expected, a fully positive community matrix.

Interestingly, also matrix J_{pos} in (21) is eventually exponentially positive. Hence, according to Theorem 10, there exists $\alpha \geq 0$ such that $J_{pos} = B - \alpha I$, with B eventually positive. Indeed, for $\alpha = 2$, $B = J_{pos} + \alpha I$ is eventually positive, irreducible and $\mathrm{index}_0(B) \leqslant 1$. Hence, in view of Theorem 8, there exists $\beta > \rho(B)$ such that $\forall\, \alpha \in (\rho(B),\, \beta)$, $\alpha I - B$ has a positive inverse: in this case, $\alpha = 2$ belongs to the interval $(\rho(B),\, \beta) = (1.91,\, 2.05)$.

7.2 Shallow lake community

The ecological network of the shallow lake community described in [21] corresponds to the interaction graph $\mathcal{G}(S)$ shown in Fig. 3(b) of the Main Paper, which is in turn associated with the sign pattern S of the community matrix J:

$$S = \begin{bmatrix} -1 & -1 & 1 & 1 & 0 & 0 \\ 1 & -1 & 0 & -1 & 0 & 0 \\ -1 & 0 & -1 & 0 & 0 & 1 \\ 0 & 0 & -1 & -1 & -1 & 1 \\ 0 & 1 & 0 & -1 & -1 & 0 \\ 0 & 0 & -1 & -1 & 0 & -1 \end{bmatrix}. \tag{22}$$

Changing sign to the first, fourth and sixth variable, namely applying the gauge transformation

$$\Sigma = \text{diag}(-1 \quad 1 \quad 1 \; -1 \quad 1 \; -1),$$

leads to the new sign pattern

$$S' = \Sigma S \Sigma = \begin{bmatrix} -1 & 1 & -1 & 1 & 0 & 0 \\ -1 & -1 & 0 & 1 & 0 & 0 \\ 1 & 0 & -1 & 0 & 0 & -1 \\ 0 & 0 & 1 & -1 & 1 & 1 \\ 0 & 1 & 0 & 1 & -1 & 0 \\ 0 & 0 & 1 & -1 & 0 & -1 \end{bmatrix}, \tag{23}$$

which corresponds to the graph in Fig. 3(c) of the Main Paper.

This new graph satisfies the assumptions of Theorem 6. Indeed, if we remove from $\mathcal{G}(S')$ all negative edges, the resulting graph $\mathcal{G}(S'^{+})$ is strongly connected. Hence, there must be a fully positive influence matrix corresponding to some choice of the parameters having the sign pattern (23).

Indeed, the choice

$$J'_{pos} = \begin{bmatrix} -0.6 & 0.6 & -0.6 & 0.6 & 0 & 0 \\ -0.6 & -0.6 & 0 & 0.6 & 0 & 0 \\ 0.6 & 0 & -0.6 & 0 & 0 & -0.6 \\ 0 & 0 & 0.6 & -1 & 0.6 & 1 \\ 0 & 0.7 & 0 & 0.6 & -1 & 0 \\ 0 & 0 & 1 & -0.6 & 0 & -0.8 \end{bmatrix} \tag{24}$$

yields $-(J'_{pos})^{-1} > 0$ elementwise.

Which is then the sign pattern of the influence matrix for the original graph? As discussed in the Main Paper, it can simply be achieved from the matrix O of all-ones by applying the same gauge transformation Σ:

$$K = \Sigma O \Sigma = \begin{bmatrix} 1 & -1 & -1 & 1 & -1 & 1 \\ -1 & 1 & 1 & -1 & 1 & -1 \\ -1 & 1 & 1 & -1 & 1 & -1 \\ 1 & -1 & -1 & 1 & -1 & 1 \\ -1 & 1 & 1 & -1 & 1 & -1 \\ 1 & -1 & -1 & 1 & -1 & 1 \end{bmatrix}.$$

Note that also J'_{pos} in (24) is eventually exponentially positive. Therefore, according to Theorem 10, there exists $\alpha \geq 0$ such that $J'_{pos} = B - \alpha I$, with B eventually positive. Indeed, for $\alpha = 1.5$, $B = J'_{pos} + \alpha I$ is eventually positive, irreducible and $\mathrm{index}_0(B) \leqslant 1$. Thus, Theorem 8 ensures that there exists $\beta > \rho(B)$ such that $\forall\ \alpha \in (\rho(B),\, \beta)$, $\alpha I - B$ has a positive inverse: in this case, $\alpha = 1.5$ clearly belongs to the interval $(\rho(B),\, \beta) = (1.47,\, 1.52)$.

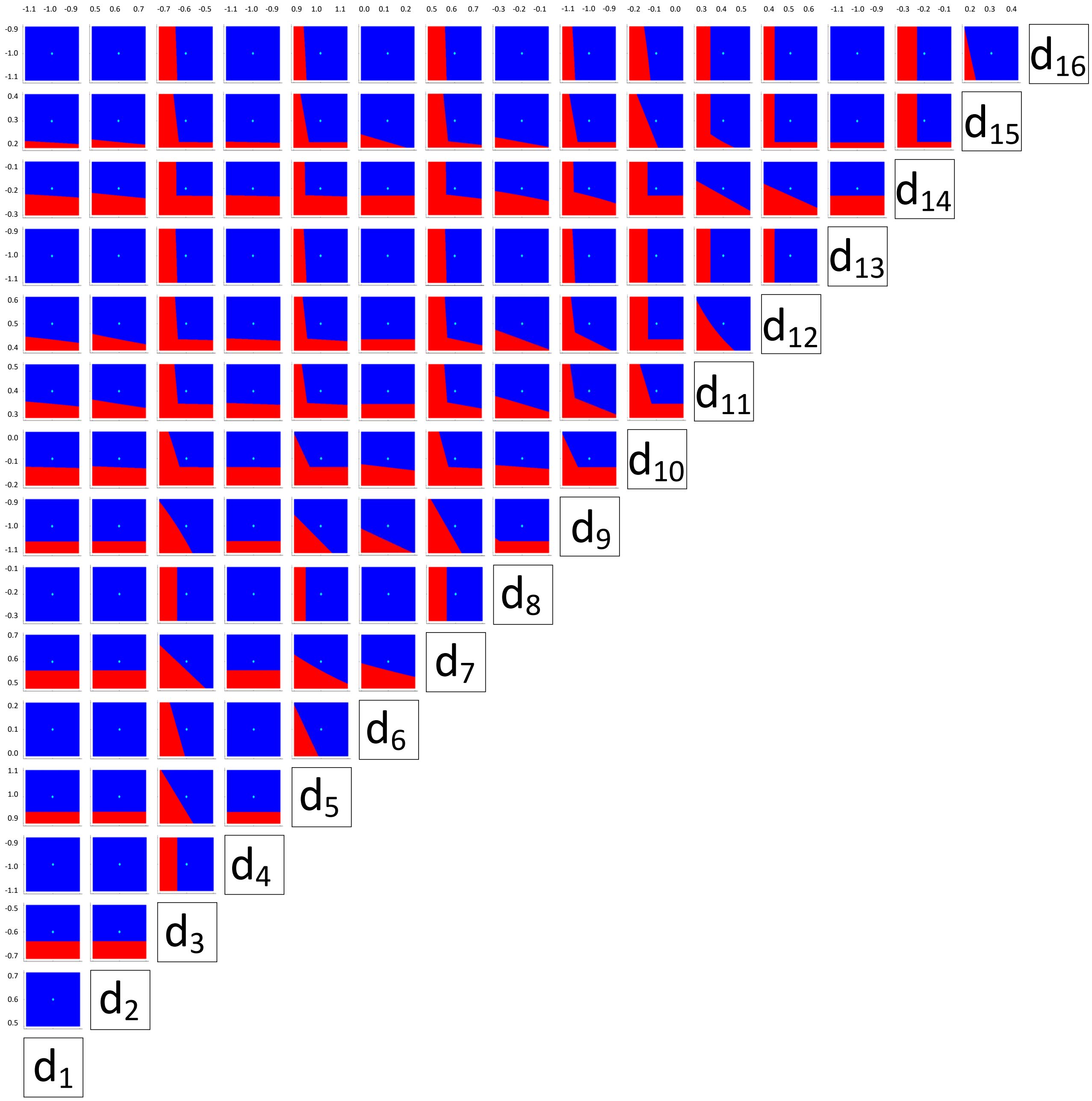

Figure 1: Phytoplankton example with nominal choice of the parameters given in (20). Sampling in the parameter space: the parameters d_i are allowed to vary in the interval between $\bar{d}_i - 0.1$ and $\bar{d}_i + 0.1$, where $\bar{d}_i$ is the nominal value. Blue points correspond to parameter choices that lead to a fully positive influence matrix, while red points correspond to parameter choices that do not lead to a fully positive influence matrix and green point correspond to parameter choices that make the community matrix singular (hence, its inverse cannot be computed). The cyan diamond in the center of each plot indicates the nominal choice of the parameters in (20).

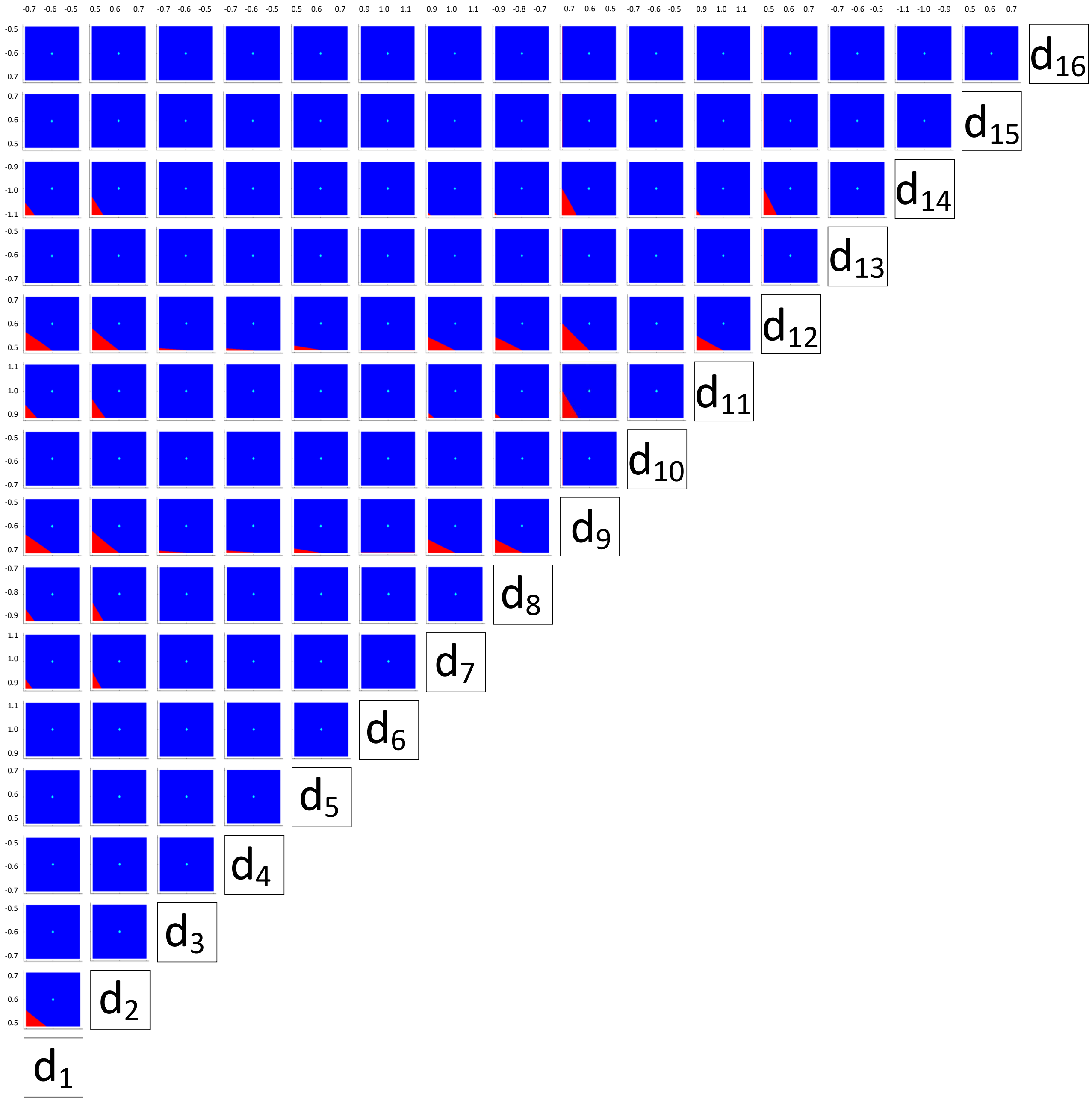

Figure 2: Phytoplankton example with nominal choice of the parameters given in (21). Sampling in the parameter space: the parameters d_i are allowed to vary in the interval between $\bar{d}_i - 0.1$ and $\bar{d}_i + 0.1$, where $\bar{d}_i$ is the nominal value. Blue points correspond to parameter choices that lead to a fully positive influence matrix, while red points correspond to parameter choices that do not lead to a fully positive influence matrix and green point correspond to parameter choices that make the community matrix singular (hence, its inverse cannot be computed). The cyan diamond in the center of each plot indicates the nominal choice of the parameters in (21).

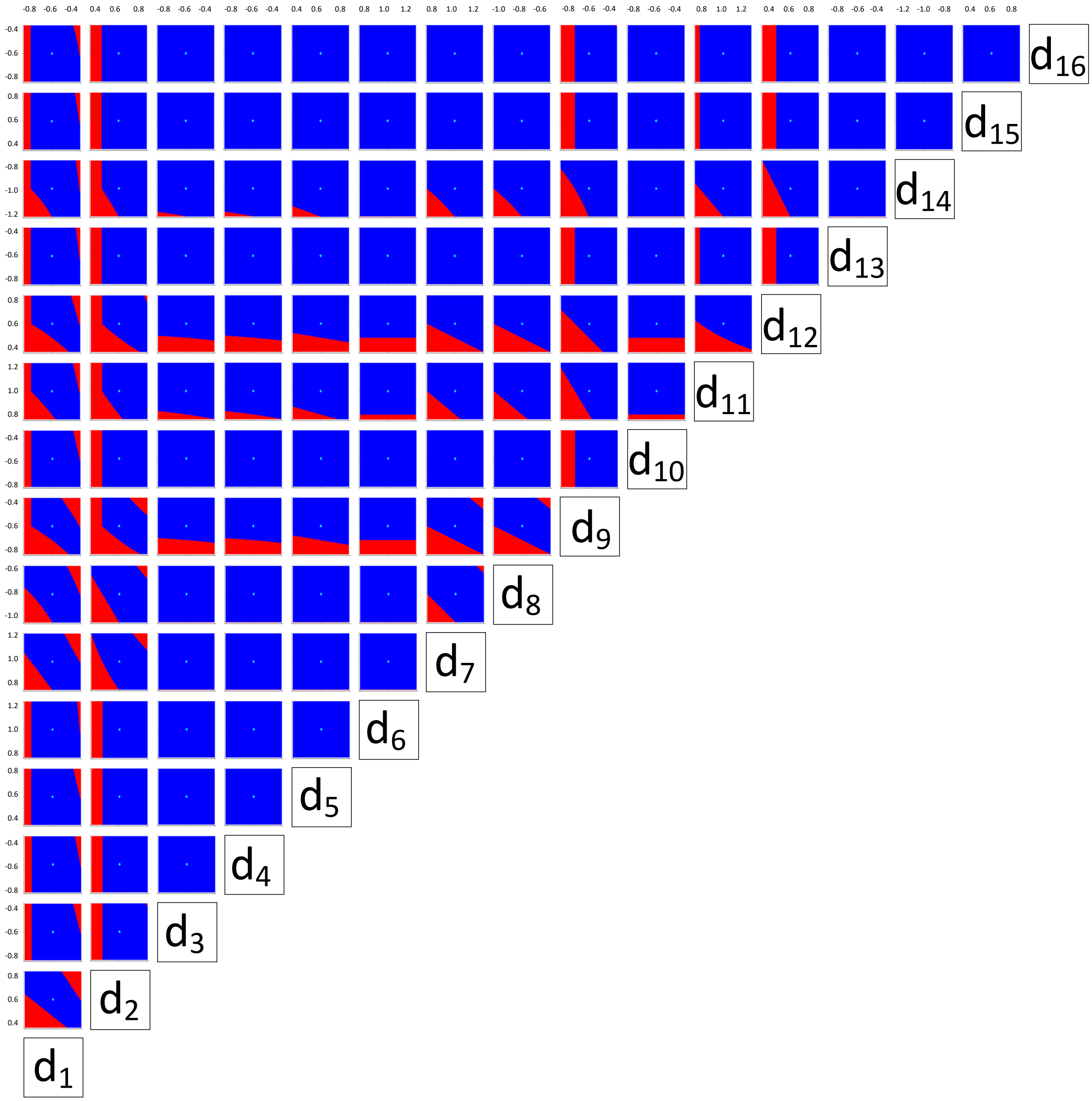

Figure 3: Phytoplankton example with nominal choice of the parameters given in (21). Sampling in the parameter space: the parameters d_i are allowed to vary in the interval between $\bar{d}_i - 0.2$ and $\bar{d}_i + 0.2$, where $\bar{d}_i$ is the nominal value. Blue points correspond to parameter choices that lead to a fully positive influence matrix, while red points correspond to parameter choices that do not lead to a fully positive influence matrix and green point correspond to parameter choices that make the community matrix singular (hence, its inverse cannot be computed). The cyan diamond in the center of each plot indicates the nominal choice of the parameters in (21).

References

[1] C. Altafini and G. Lini. Predictable dynamics of opinion forming for networks with antagonistic interactions. *IEEE Transactions on Automatic Control*, 60(2):342–357, Feb 2015.

[2] E. A. Bender, T. J. Case, and M. E. Gilpin. Perturbation experiments in community ecology: Theory and practice. *Ecology*, 65(1):1–13, 1984.

[3] A. Berman, M. Catral, L. M. Dealba, A. Elhashash, F. J. Hall, L. Hogben, I.-J. Kim, D. D. Olesky, P. Tarazaga, M. J. Tsatsomeros, and P. van den Driessche. Sign patterns that allow eventual positivity. *ELA. The Electronic Journal of Linear Algebra [electronic only]*, 19:108–120, 2009.

[4] A. Berman and R. Plemmons. *Nonnegative Matrices in the Mathematical Sciences*. Number pt. 11 in Classics in Applied Mathematics. Society for Industrial and Applied Mathematics, 1994.

[5] F. Blanchini, E. Franco, and G. Giordano. Determining the structural properties of a class of biological models. In *Proceedings of the IEEE Conference on Decision and Control*, pages 5505–5510, Maui (HI), USA, 2012.

[6] F. Blanchini and G. Giordano. Piecewise-linear Lyapunov functions for structural stability of biochemical networks. *Automatica*, 50(10):2482–2493, 2014.

[7] R. Brualdi and B. Shader. *Matrices of Sign-Solvable Linear Systems*. Cambridge Univ. Press, 1995.

[8] J. Dambacher, R. Levins, and P. Rossignol. Life expectancy change in perturbed communities: Derivation and qualitative analysis. *Mathematical Biosciences*, 197(1):1–14, SEP 2005.

[9] J. Dambacher, H. Li, and P. Rossignol. Relevance of community structure in assessing indeterminacy of ecological predictions. *Ecology*, 83(5):1372–1385, 2002.

[10] J. Dambacher, H. Li, and P. Rossignol. Qualitative predictions in model ecosystems. *Ecological Modelling*, 161(1-2):79–93, MAR 2003.

[11] J. M. Dambacher, H.-K. Luh, H. W. Li, and P. A. Rossignol. Qualitative stability and ambiguity in model ecosystems. *The American Naturalist*, 161(6):876–888, 2003.

[12] J. M. Dambacher and R. Ramos Jiliberto. Understanding and predicting effects of modified interactions through a qualitative analysis of community structure. *The Quarterly Review of Biology*, 82(3):227–250, 2007.

[13] T. Drengstig, H. R. Ueda, and P. Ruoff. Predicting perfect adaptation motifs in reaction kinetic networks. *The Journal of Physical Chemistry B*, 112(51):16752–16758, 2008.

[14] G. Facchetti, G. Iacono, and C. Altafini. Computing global structural balance in large-scale signed social networks. *PNAS*, 108(52):20953–20958, 2011.

[15] L. Farina and S. Rinaldi. *Positive Linear Systems: Theory and Applications*. Wiley, New York, 2000.

[16] M. Fiedler and R. Grone. Characterizations of sign patterns of inverse-positive matrices. *Linear Algebra and its Applications*, 40:237 – 245, 1981.

[17] F. R. Gantmacher. *Applications of the theory of matrices*. Interscience, New York, 1959.

[18] G. Giordano. *Structural Analysis and Control of Dynamical Networks*. PhD thesis, Università degli Studi di Udine, 2016.

[19] G. Giordano, C. Cuba Samaniego, E. Franco, and F. Blanchini. Computing the structural influence matrix for biological systems. *Journal of Mathematical Biology*, 72(7):1927–1958, 2016.

[20] F. Golnaraghi and B. C. Kuo. *Automatic Control Systems (9th ed.)*. John Wiley & Sons, 2009.

[21] G. R. Hosack, H. W. Li, and P. A. Rossignol. Sensitivity of system stability to model structure. *Ecological Modelling*, 220(8):1054 – 1062, 2009.

[22] A. C. Iles and M. Novak. Complexity increases predictability in allometrically constrained food webs. *The American Naturalist*, 188(1):87–98, 2016.

[23] D. Koslicki and M. Novak. Exact probabilities for the indeterminacy of complex networks as perceived through press perturbations. *arXiv:1610.07705v1*, 2016.

[24] H. T. Le and J. J. McDonald. Inverses of M-type matrices created with irreducible eventually nonnegative matrices. *Linear Algebra and its Applications*, 419:668 – 674, 2006.

[25] R. Levins. *Evolution in changing environments: some theoretical explorations*. Princeton University Press, 1968.

[26] R. Levins. The qualitative analysis of partially specified systems. *Annals of the New York Academy of Science*, 231:123–138, 1974.

[27] R. Levins. Evolution in communities near equilibrium. In M. Cody and J. M. Diamond, editors, *Ecology and evolution of communities*, pages 16–50. Harvard University Press, 1975.

[28] R. M. May. *Stability and Complexity in Model Ecosystems, 2nd ed.* Princeton University Press, 1974.

[29] J. M. Montoya, G. Woodward, M. C. Emmerson, and R. V. Sol. Press perturbations and indirect effects in real food webs. *Ecology*, 90(9):2426–2433, 2009.

[30] D. Noutsos. On Perron-Frobenius property of matrices having some negative entries. *Linear Algebra and its Applications*, 412:132 – 153, 2006.

[31] D. Noutsos and M. J. Tsatsomeros. Reachability and holdability of nonnegative states. *SIAM Journal on Matrix Analysis and Applications*, 30(2):700–712, 2008.

[32] M. Novak, J. Yeakel, A. E. Noble, D. F. Doak, M. Emmerson, J. A. Estes, U. Jacob, M. T. Tinker, and J. T. Wootton. Characterizing species interactions to understand press perturbations: What is the community matrix? *Annual Review of Ecology, Evolution, and Systematics* 47:409–432, 2016.

[33] M. Novak, J. T. Wootton, D. F. Doak, M. Emmerson, J. A. Estes, and M. T. Tinker. Predicting community responses to perturbations in the face of imperfect knowledge and network complexity. *Ecology*, 92(4):836–846, 2011.

[34] D. D. Olesky, M. J. Tsatsomeros, and P. van den Driessche. M_v matrices: a generalization of m-matrices based on eventually nonnegative matrices. *Electronic Journal of Linear Algebra*, 18:339351, 2009.

[35] O. J. Schmitz. Press perturbations and the predictability ofecological interactions in a food web. *Ecology*, 78(1):55–69, 1997.

[36] H. L. Smith. Systems of ordinary differential equations which generate an order preserving flow. A survey of results. *SIAM Rev.*, 30(1):87–113, 1988.

[37] H. L. Smith. *Monotone Dynamical Systems: An Introduction to the Theory of Competitive and Cooperative Systems*, volume 41 of *Mathematical Surveys and Monographs*. AMS, Providence, RI, 1995.

[38] E. Sontag. Adaptation and regulation with signal detection implies internal model. *Systems & Control Letters*, 50(2):119–126, 2003.

[39] E. D. Sontag. Monotone and near-monotone biochemical networks. *Syst. Synth. Biol.*, 1(2):59–87, 2007.

[40] L. Stone. Phytoplankton-bacteria-protozoa interactions: a qualitative model portraying indirect effects. *Marine Ecology Progress Series*, 64(1-2):137 – 145, 1990.

[41] T. M. Yi, Y. Huang, M. I. Simon, and J. Doyle. Robust perfect adaptation in bacterial chemotaxis through integral feedback control. *Proceedings of the National Academy of Sciences of the USA*, 97(9):4649–4653, 2000.

[42] P. Yodzis. The indeterminacy of ecological interactions as perceived through perturbation experiments. *Ecology*, 69(2):508–515, 1988.
